# SLCO5A1 and synaptic assembly genes contribute to impulsivity in juvenile myoclonic epilepsy

**DOI:** 10.1038/s41525-023-00370-z

**Published:** 2023-09-28

**Authors:** Delnaz Roshandel, Eric J. Sanders, Amy Shakeshaft, Naim Panjwani, Fan Lin, Amber Collingwood, Anna Hall, Katherine Keenan, Celine Deneubourg, Filippo Mirabella, Simon Topp, Jana Zarubova, Rhys H. Thomas, Inga Talvik, Marte Syvertsen, Pasquale Striano, Anna B. Smith, Kaja K. Selmer, Guido Rubboli, Alessandro Orsini, Ching Ching Ng, Rikke S. Møller, Kheng Seang Lim, Khalid Hamandi, David A. Greenberg, Joanna Gesche, Elena Gardella, Choong Yi Fong, Christoph P. Beier, Danielle M. Andrade, Heinz Jungbluth, Mark P. Richardson, Annalisa Pastore, Manolis Fanto, Deb K. Pal, Lisa J. Strug, the BIOJUME Consortium, Zuzana Šobíšková, Cechovaz Pracoviste, Michaela Kajsova, Rikke S. Møller, Elena Gardella, Maria Miranda, Pasquale Striano, Alessandro Orsini, Pronab Bala, Amy Kitching, Kate Irwin, Lorna Walding, Lynsey Adams, Uma Jegathasan, Rachel Swingler, Rachel Wane, Julia Aram, Nikil Sudarsan, Dee Mullan, Rebecca Ramsay, Vivien Richmond, Mark Sargent, Paul Frattaroli, Matthew Taylor, Marie Home, Sal Uka, Susan Kilroy, Tonicha Nortcliffe, Halima Salim, Kelly Holroyd, Alison McQueen, Dympna Mcaleer, Dina Jayachandran, Dawn Egginton, Bridget MacDonald, Michael Chang, David Deekollu, Alok Gaurav, Caroline Hamilton, Jaya Natarajan, Inyan Takon, Janet Cotta, Nick Moran, Jeremy Bland, Rosemary Belderbos, Heather Collier, Joanne Henry, Matthew Milner, Sam White, Michalis Koutroumanidis, William Stern, Jennifer Quirk, Javier Peña Ceballos, Anastasia Papathanasiou, Ioannis Stavropoulos, Dora Lozsadi, Andrew Swain, Charlotte Quamina, Jennifer Crooks, Tahir Majeed, Sonia Raj, Shakeelah Patel, Michael Young, Melissa Maguire, Munni Ray, Caroline Peacey, Linetty Makawa, Asyah Chhibda, Eve Sacre, Shanaz Begum, Martin O’ Malley, Lap Yeung, Claire Holliday, Louise Woodhead, Karen Rhodes, Shan Ellawela, Joanne Glenton, Verity Calder, John Davis, Paul McAlinden, Sarah Francis, Lisa Robson, Karen Lanyon, Graham Mackay, Elma Stephen, Coleen Thow, Margaret Connon, Martin Kirkpatrick, Susan MacFarlane, Anne Macleod, Debbie Rice, Siva Kumar, Carolyn Campbell, Vicky Collins, William Whitehouse, Christina Giavasi, Boyanka Petrova, Thomas Brown, Catie Picton, Michael O’Donoghue, Charlotte West, Helen Navarra, Seán J. Slaght, Catherine Edwards, Andrew Gribbin, Liz Nelson, Stephen Warriner, Heather Angus-Leppan, Loveth Ehiorobo, Bintou Camara, Tinashe Samakomva, Rajiv Mohanraj, Vicky Parker, Rajesh Pandey, Lisa Charles, Catherine Cotter, Archana Desurkar, Alison Hyde, Rachel Harrison, Markus Reuber, Rosie Clegg, Jo Sidebottom, Mayeth Recto, Patrick Easton, Charlotte Waite, Alice Howell, Jacqueline Smith, Shyam Mariguddi, Zena Haslam, Elizabeth Galizia, Hannah Cock, Mark Mencias, Samantha Truscott, Deirdre Daly, Hilda Mhandu, Nooria Said, Mark Rees, Seo-Kyung Chung, Owen Pickrell, Beata Fonferko-Shadrach, Mark Baker, Fraser Scott, Naveed Ghaus, Gail Castle, Jacqui Bartholomew, Ann Needle, Julie Ball, Andrea Clough, Shashikiran Sastry, Charlotte Busby, Amit Agrawal, Debbie Dickerson, Almu Duran, Muhammad Khan, Laura Thrasyvoulou, Eve Irvine, Sarah Tittensor, Jacqueline Daglish, Sumant Kumar, Claire Backhouse, Claire Mewies, Julia Aram, Nikil Sudarsan, Dee Mullan, Rebecca Ramsay, Vivien Richmond, Denise Skinner, Mark Sargent, Rahul Bharat, Sarah-Jane Sharman, Arun Saraswatula, Helen Cockerill

**Affiliations:** 1 Genetics and Genome Biology Program, The Hospital for Sick Children, Toronto, Canada; 2 Division of Biostatistics, Dalla Lana School of Public Health, The University of Toronto, Toronto, Canada; 3 Department of Basic & Clinical Neurosciences, Institute of Psychiatry, Psychology & Neuroscience, King’s College London, London, UK; 4 MRC Centre for Neurodevelopmental Disorders, King’s College London, London, UK; 5 Department of Neurology, Second Faculty of Medicine, Charles University and Motol University Hospital, Prague, Czech Republic; 6 Newcastle upon Tyne NHS Foundation Trust, Newcastle, UK; 7 Translational and Clinical Research Institute, Faculty of Medical Sciences, Newcastle University, Newcastle, UK; 8 Tallin Children’s Hospital, Tallin, Estonia; 9 Department of Neurology, Drammen Hospital, Vestre Viken Health Trust, Oslo, Norway; 10 IRCCS Istituto ‘G. Gaslini’, Genova, Italy; 11 Department of Neurosciences, Rehabilitation, Ophthalmology, Genetics, Maternal and Child Health, University of Genova, Genova, Italy; 12 Department of Research and Innovation, Division of Clinical Neuroscience, Oslo University Hospital, Oslo, Norway; 13 National Centre for Epilepsy, Oslo University Hospital, Oslo, Norway; 14 Danish Epilepsy Centre, Dianalund, Denmark; 15 University of Copenhagen, Copenhagen, Denmark; 16 Pediatric Neurology, Azienda Ospedaliero-Universitaria Pisana, Pisa University Hospital, Pisa, Italy; 17 Institute of Biological Sciences, Faculty of Science, University of Malaya, Kuala Lumpur, Malaysia; 18 Department of Regional Health Research, University of Southern Denmark, Odense, Denmark; 19 Division of Neurology, Department of Medicine, Faculty of Medicine, University of Malaya, Kuala Lumpur, Malaysia; 20 The Welsh Epilepsy Unit, Department of Neurology Cardiff & Vale University Health Board, Cardiff, UK; 21 Department of Psychological Medicine and Clinical Neuroscience, Cardiff University, Cardiff, UK; 22 Nationwide Children’s Hospital, Columbus, OH USA; 23 Odense University Hospital, Odense, Denmark; 24 Division of Paediatric Neurology, Department of Pediatrics, Faculty of Medicine, University of Malaya, Kuala Lumpur, Malaysia; 25 Adult Epilepsy Genetics Program, Krembil Research Institute, University of Toronto, Toronto, Canada; 26 Randall Centre for Cell and Molecular Biophysics, Muscle Signalling Section, Faculty of Life Sciences and Medicine, King’s College London, London, UK; 27 Department of Paediatric Neurology, Neuromuscular Service, Evelina’s Children Hospital, Guy’s & St. Thomas’ Hospital NHS Foundation Trust, London, UK; 28 King’s College Hospital, London, UK; 29 Departments of Statistical Sciences and Computer Science, The University of Toronto, Toronto, Canada; 30 The Centre for Applied Genomics, The Hospital for Sick Children, Toronto, Canada; 31 Airedale NHS Foundation Trust, Keighley, UK; 32 Ashford and St. Peter’s Hospitals NHS Foundation Trust, Chertsey, UK; 33 Bradford Teaching Hospitals NHS Foundation Trust, Bradford, UK; 34 Brighton and Sussex University Hospitals NHS Trust, Brighton, UK; 35 Calderdale and Huddersfield Foundation Trust, Huddersfield, UK; 36 County Durham and Darlington NHS Foundation Trust, Darlington, UK; 37 Croydon Health Services NHS Trust, Croydon, UK; 38 Cwm Taf Morgannwg University Health Board, Mountain Ash, UK; 39 East and North Hertfordshire NHS Trust, Stevenage, UK; 40 East Kent Hospitals University NHS Foundation Trust, Canterbury, UK; 41 East Lancashire Hospitals NHS Trust, Burnley, UK; 42 Guy’s and St Thomas’ NHS Foundation Trust, London, UK; 43 Kingston Hospital NHS Foundation Trust, Kingston upon Thames, UK; 44 Lancashire Teaching Hospitals NHS Foundation Trust, Preston, UK; 45 Leeds Teaching Hospitals NHS Trust, Leeds, UK; 46 Manchester University NHS Foundation Trust, Manchester, UK; 47 NHS Grampian, Aberdeen, UK; 48 NHS Tayside, Dundee, UK; 49 North Tees and Hartlepool NHS Foundation Trust, Stockton-on-Tees, UK; 50 Nottingham University Hospitals NHS Trust, Nottingham, UK; 51 Portsmouth Hospitals NHS Trust, Portsmouth, UK; 52 Royal Free London NHS Foundation Trust, London, UK; 53 Salford Royal NHS Foundation Trust, Salford, UK; 54 Sandwell & West Birmingham Hospitals NHS Trust, Birmingham, UK; 55 Sheffield Children’s NHS Foundation Trust, Sheffield, UK; 56 Sheffield Teaching Hospitals NHS Foundation Trust, Sheffield, UK; 57 Southport and Ormskirk Hospital NHS Trust, Southport, UK; 58 St George’s University Hospitals NHS Foundation Trust, London, UK; 59 Swansea University Medical School and Swansea Bay University Health board, Swansea, UK; 60 The Mid Yorkshire Hospitals NHS Trust, Wakefield, UK; 61 The Royal Wolverhampton NHS Trust, Wolverhampton, UK; 62 The Walton Centre NHS Foundation Trust, Liverpool, UK; 63 University Hospitals Birmingham NHS Foundation Trust, Birmingham, London, UK; 64 University Hospitals of Derby and Burton NHS Foundation Trust, Derby, UK; 65 University Hospitals Sussex Hospitals NHS Foundation Trust, Brighton, UK; 66 University Hospitals Plymouth NHS Trust, Plymouth, UK; 67 West Suffolk NHS Foundation Trust, Bury Saint Edmunds, UK

**Keywords:** Genetics research, Epilepsy

## Abstract

Elevated impulsivity is a key component of attention-deficit hyperactivity disorder (ADHD), bipolar disorder and juvenile myoclonic epilepsy (JME). We performed a genome-wide association, colocalization, polygenic risk score, and pathway analysis of impulsivity in JME (*n* = 381). Results were followed up with functional characterisation using a drosophila model. We identified genome-wide associated SNPs at 8q13.3 (*P* = 7.5 × 10^−9^) and 10p11.21 (*P* = 3.6 × 10^−8^). The 8q13.3 locus colocalizes with *SLCO5A1* expression quantitative trait loci in cerebral cortex (*P* = 9.5 × 10^−3^). *SLCO5A1* codes for an organic anion transporter and upregulates synapse assembly/organisation genes. Pathway analysis demonstrates 12.7-fold enrichment for presynaptic membrane assembly genes (*P* = 0.0005) and 14.3-fold enrichment for presynaptic organisation genes (*P* = 0.0005) including *NLGN1* and *PTPRD*. RNAi knockdown of *Oatp30B*, the *Drosophila* polypeptide with the highest homology to *SLCO5A1*, causes over-reactive startling behaviour (*P* = 8.7 × 10^−3^) and increased seizure-like events (*P* = 6.8 × 10^−7^). Polygenic risk score for ADHD genetically correlates with impulsivity scores in JME (*P* = 1.60 × 10^−3^). *SLCO5A1* loss-of-function represents an impulsivity and seizure mechanism. Synaptic assembly genes may inform the aetiology of impulsivity in health and disease.

## Introduction

Impulsivity is a heritable behavioural trait leading to actions that are “poorly conceived, prematurely expressed, unduly risky, or inappropriate to the situation and that often result in undesirable consequences”^1^. Estimates of heritability for impulsivity from a study of twins were between 33% and 56% at ages 11–13 years, and between 19% and 44% at ages 14–16^2^. Raised impulsivity is a key endophenotype of attention-deficit hyperactivity disorder (ADHD)^3^, bipolar disorder^4^ and juvenile myoclonic epilepsy (JME)^5–7^. ADHD is characterised by inattention, hyperactivity and impulsivity. Individuals with ADHD show more signs of impulsivity (attentional, non-planning and motor) compared to controls^8^. A previous genome-wide association study (GWAS) of impulsive personality traits (UPPS-P Sensation Seeking, Drug Experimentation and UPPS-P Negative Urgency) in 22,861 healthy individuals of European ancestry demonstrated two significant associated loci at 3p12.1 and 22q13.1^9^. Variants at the 3p12.1 locus correlated with predicted *Cell Adhesion Molecule–2* (*CADM2*) expression, in the putamen^10^, and the 22q13.1 locus near *CACNA1I* has been previously implicated in schizophrenia^11^. CADM2 mediates synaptic signalling and is highly expressed in the human cerebral cortex and cerebellum^12^. Given impulsivity is elevated in neuropsychiatric disorders, there may be shared genetic mechanisms across disorders and/or with impulsivity in the general population, however to our knowledge there has been no GWAS of impulsivity in any neuropsychiatric disorder.

Impulsivity is elevated in different epilepsies, but the evidence across multiple dimensions of impulsivity is strongest in JME^5–7^. JME is a common adolescent-onset syndrome characterised by awakening myoclonic, generalised tonic-clonic and absence seizures, often triggered by sleep deprivation. Trait impulsivity in JME is associated with the frequency of both myoclonic and absence seizures^6^, but it is not clear if this indicates a causal relationship or a common mechanism regulating both impulsivity and seizures, though convergent lines of evidence suggest the involvement of overlapping prefrontal-striatal networks in both JME and impulsivity^13–20^. Finding a shared aetiology would offer new therapeutic approaches for drug-resistant epilepsy.

The overall syndrome of JME has complex inheritance with few replicated susceptibility loci^21,22^, and other loci with less support^22–24^. A major challenge for epilepsies of complex inheritance is to explain the wide variation in phenotypic expression and treatment response between individuals. Forty-percent experience antiseizure medication (ASM) resistance or intolerance^25^. In addition, no current ASM modifies the lifelong disease course of JME and the pharmacological options are sparse, especially for women^25^. Hence novel treatments based on genetic disease mechanisms, such as those emerging for monogenic channelopathy and mTOR pathway epilepsies, are urgently needed^26,27^. Our methodological approach is to carry out genome-wide analysis of endophenotypes in JME such as impulsivity and clinically relevant outcomes such as ASM resistance, a strategy with predicted advantages for reducing heterogeneity, increasing statistical power^28,29^ and improving direct clinical translation for precision medicine.

## Results

### Genome-wide association analysis with BIS-Brief

**Fig. 1:**
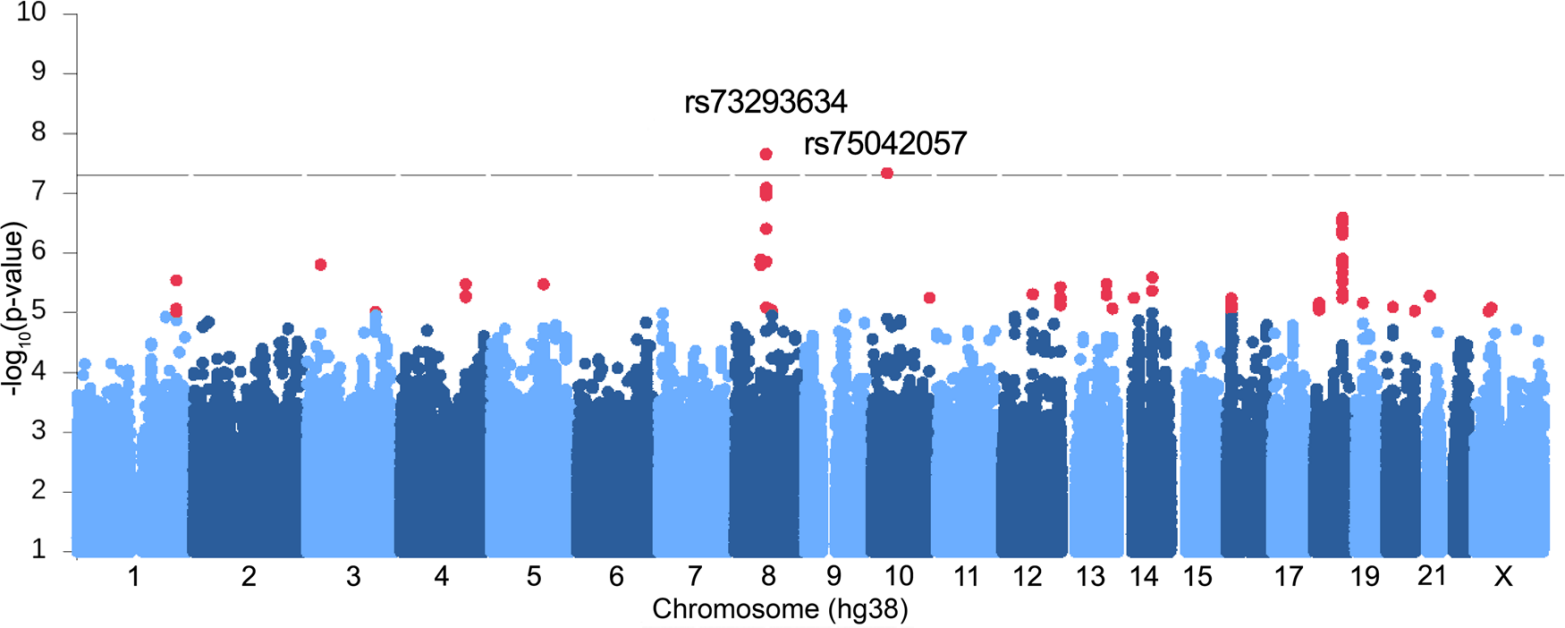
Manhattan plot showing GWAS with BIS-Brief score. Linear regression was used to test association of each SNP with BIS-Brief. Sex, genotyping batch, age at consent, first three PCs, and the frequency of myoclonus or absence seizures were included as covariates in the model. We found two significant genome-wide associations on chromosome 8 (rs73293634 (G/T)) and 10 (rs75042057 (T/G)) in the analysis of 324 European individuals with JME. Variants below −log_10_*P* < 1 were omitted in the plot.

We investigated the influence of 8,950,360 variants on impulsivity in European ancestry patients with JME (*n* = 324) and a mega-analysis including all ancestries (*n* = 372), who self-rated their trait impulsivity using the Barratt Impulsivity Scale, eight-item BIS-Brief version^30^. We conducted a GWAS of BIS-Brief score (Supplementary Fig. 2) in the European subset, adjusted for sex, genotyping batch, age at consent, population stratification, and seizure frequency (Supplementary Table 2). We discovered two genome-wide significant loci, one on chromosome 8 (rs73293634 (G/T)) and one on chromosome 10 (rs75042057 (T/G) (Fig. 1, Table 1, Supplementary Figs. 3 and 4). Given the distribution of BIS-Brief was slightly right skewed, for sensitivity analysis we tested the SNP associations using an inverse rank normal transformed BIS-Brief phenotype as well. Qualitatively similar results were obtained with rs73293634 and rs75042057 demonstrating association with *p* = 3.1 × 10^−8^ and *p* = 1.4 × 10^−7^, respectively (Supplementary Table 3). The distribution of BIS-Brief by rs73293634 and rs75042057 genotypes are provided in Supplementary Fig. 5. In a mega-analysis comprised of all ancestral groups (Supplementary Fig. 6), these loci were further supported including by a nearby chromosome 8 SNP (rs146866040, *r*^*2*^ = 0.89) with stronger evidence of association in the combined ancestry mega-analysis as measured by the *p* value (*P* = 1.57 × 10^−9^; Table 1), providing cross-ancestral support for the locus.

**Table 1:** Summary of genome-wide associated variants for the GWAS of BIS scores in JME (*n* = 324).

		European GWAS (*n* = 324)				Mega-GWAS (*n* = 372)			
Variant ID (hg38)	Imputation *r*^2^	MAF	Beta	SE	*P* value	MAF	Beta	SE	*P* value
chr8:69,884,968* rs73293634 (G/T)	0.961	0.036 (T)	5.42	0.91	7.5 × 10^−9^	0.041	4.55	0.79	1.61 × 10^−8^
chr8:69,876,965 rs146866040 (A/G)	0.979	0.032 (G)	5.38	0.94	2.5 × 10^−8^	0.031	5.60	0.90	1.57 × 10^−9^
chr10:34,202,650 rs75042057 (T/G)	0.878	0.019 (G)	7.51	1.33	3.6 × 10^−8^	0.022	6.60	1.19	4.99 × 10^−8^

Linear regression was used to test association of each SNP with BIS-Brief. Sex, genotyping batch, age at consent, first 3 PCs, and the frequency of myoclonus or absence seizures were included as covariates in the model in the European analysis. Sex, genotyping batch, and population stratification were included as covariates in the mega-GWAS.

All observed sample allele frequencies are comparable to those seen in the European 1000 Genomes (phase 3)^60^.

*The lead SNP for the mega-GWAS was rs146866040. The LD between them is *r*^2^ = 0.89 or *D*’ = 1.0.

rs73293634 falls in an intergenic region near *SLCO5A1*. The phenotypic variation explained (PVE) for rs73293634 was 10.1% in the European analysis. Although a second JME cohort with impulsivity measured is not available for replication, Watanabe et al. ^31^ reported a rs73293634 association with risk taking in the UK Biobank, where they asked the question “Would you describe yourself as someone who takes risks?” (OR (95% CI) = 1.032 (1.001–1.065), *p* = 0.04, minor allele frequency (MAF) = 0.03, *N* = 371,049). Association results posted on the same data by the Neale Lab^32^ with ~23 K fewer participants, provides a similar qualitative conclusion (*β* (SE) = 0.005 (0.003), *p* = 0.09, MAF = 0.03, *N* = 348,549). Two individuals with large structural deletions that include *SLCO5A1* are reported in the Decipher Genomics database with seizures and neurodevelopmental disorder (www.deciphergenomics.org/gene/SLCO5A1/patient-overlap/cnvs).

The significant genome-wide association on chromosome 10 (rs75042057) falls in intron 22 of *PARD3* (NM_001184785.2). The PVE by the SNP is 9.3%, although there are no variants in linkage disequilibrium with this SNP so further interrogation and confirmation of this locus is required. We note, however, that significant linkage (multipoint max LOD 4.23, alpha 0.34) was previously reported to this locus in French-Canadian families with idiopathic generalised epilepsy (IGE)^33^, of which JME is a common subtype. As well, rs75042057 was also associated with risk-taking in the UK Biobank (OR (95% CI) = 1.067 (1.029–1.106), *p* = 4.79E-4, MAF = 0.02, *N* = 371,049)^31^.

### Colocalization analysis with gene expression

**Fig. 2:**
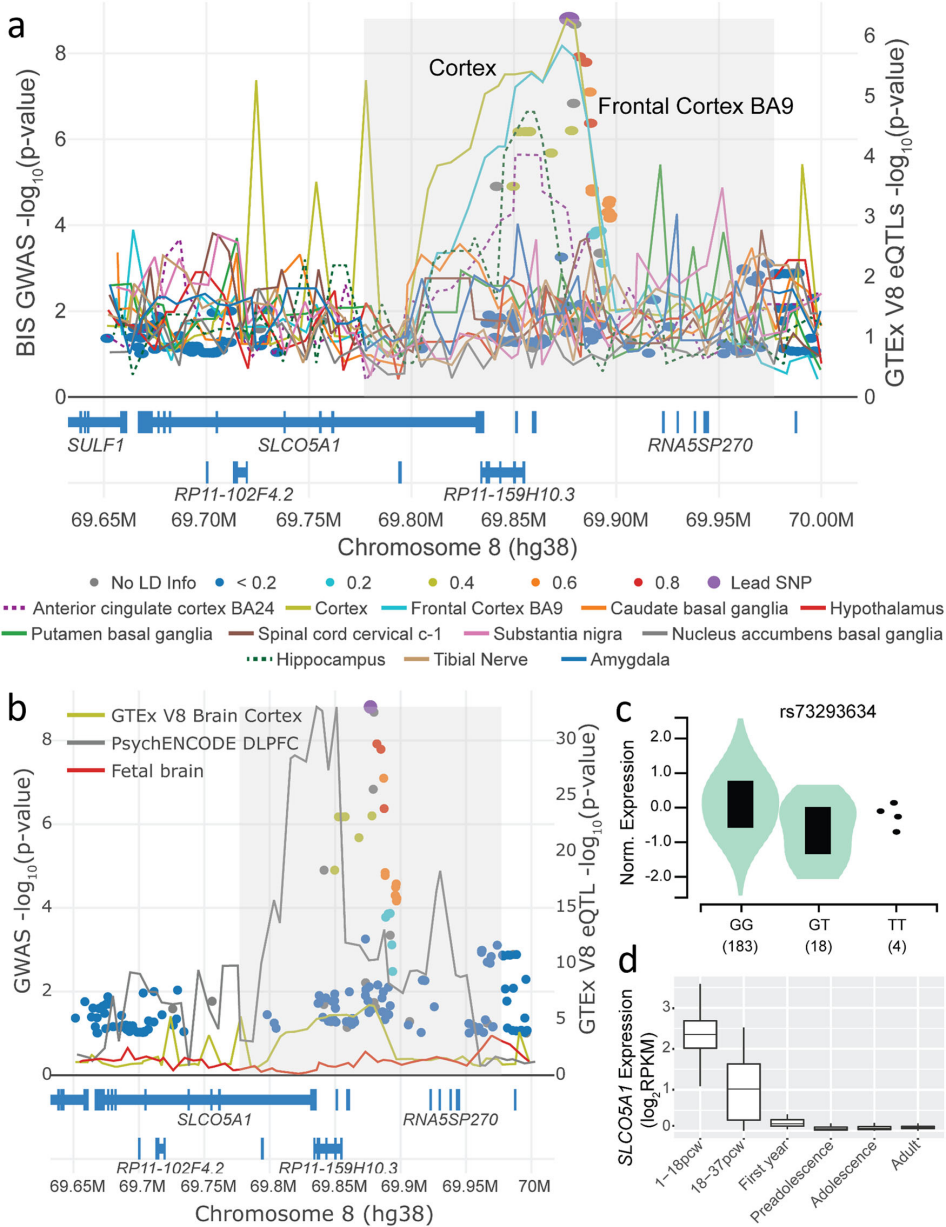
LocusFocus^70^ plot for the GWAS with BIS-Brief in JME (circles) and eQTLs in GTEx^12^ brain and tibial nerve tissues for the *SLCO5A1* gene (lines). The Simple Sum 2^36^ and COLOC2^69^ colocalization methods implemented in LocusFocus (v1.4.9)^70^ were used to test for colocalization of the BIS-Brief genome-wide peaks with eQTL analyses brain tissues from GTEx v8^12^, PsychENCODE^34^, and fetal brain^35^. **a** Colocalization figure from LocusFocus for the *SLCO5A1* gene. Lines depict the minimum *P* value trace in a sliding window for *SLCO5A1* eQTLs from GTEx, one line per tissue. Circles depict the GWAS with BIS-Brief, with the lead SNP in purple and pairwise LD with the lead SNP marked as shown in the legend, calculated using the 1000 Genomes Project^60^ European subset. Significant colocalization is observed for *SLCO5A1* eQTLs in GTEx v8 for the cerebral cortex after increasing sample size in a mega-GWAS (*n* = 367, −log_10_ *Simple Sum 2*^36 ^*P* = 9.5 × 10^−3^). Colocalization analysis with only the Europeans is provided in Supplementary Fig. 7. Colocalization was also tested for all other nearby genes shown in the figure, but no other genes’ eQTLs colocalized with BIS-Brief GWAS (not shown). **b** Colocalization analysis with PsychENCODE eQTLs in the dorsolateral prefrontal cortex (DLPFC) (*n* = 1866)^34^, and eQTLs derived from second trimester fetal brains (*n* = 120)^35^, with GTEx’s brain cortex eQTL as in A provided for reference. Colocalization analysis results suggest no colocalization with either PsychENCODE (*Simple Sum 2* *P* = 0.985) or fetal brain eQTLs (does not pass first stage test in Simple Sum 2 for having significant eQTLs in the region). **c** Violin plot for the eQTL effect of rs73293634 SNP on *SLCO5A1* expression in the cerebral cortex from GTEx v8. **d** Expression change of *SLCO5A1* from brains in various developmental stages from BrainSpan^37,38^. pcw, post conception weeks; preadolescence, 2–12 years old (inclusive); adolescence, 13–19 years old; adult, ≥20 years old (oldest samples are 40 years old). The centre lines represent the 50th percentile (median) and the bounds of the boxes are the 75th and 25th percentiles (interquartile range) with the whiskers being the largest value within 1.5 times the interquartile range above the 75th percentile and smallest values within 1.5 times the interquartile range below the 25th percentile.

Since the GWAS-associated variants are not exonic, we next assessed whether the variants impact gene expression, and for which gene in which tissue of origin, by assessing colocalization of the genome-wide significant peaks with expression quantitative trait loci (eQTL) in brain tissues. We used eQTLs from the Genotype-Tissue Expression project (GTEx) v8^12^, PsychENCODE^34^, and human fetal brains^35^ and combined them with the GWAS summary statistics from the mega-analysis, for colocalization analysis adjusting for multiple hypothesis testing^36^. Colocalization analysis with eQTLs from GTEx brain and tibial nerve tissues for genes at the locus (chr8:69,650,000–70,000,000, hg38) shows significant colocalization with *SLCO5A1* in the cerebral cortex, and no colocalization with other genes in the region (Fig. 2a and Supplementary Fig. 7; Simple Sum 2 colocalization *P* = 9.5 × 10^−3^). The minor allele for the lead SNP rs73293634 (T) decreases expression in GTEx cerebral cortex (Fig. 2c). We found no significant colocalization with eQTLs from PsychENCODE^34^ and fetal brains^35^, although nearby variants in the locus in adult brains in PsychENCODE have, in general, a clear influence on *SLCO5A1* expression (Fig. 2b). According to BrainSpan^37,38^, *SLCO5A1* is highly expressed prenatally, with expression dropping after birth but remains detectable throughout adulthood (Fig. 2d). We did not observe significant colocalization at the chromosome 10 locus with eQTLs from adult brains in GTEx^12^, PsychENCODE^34^ or fetal brains^35^.

### Functional characterisation of *SLCO5A1*

**Fig. 3:**
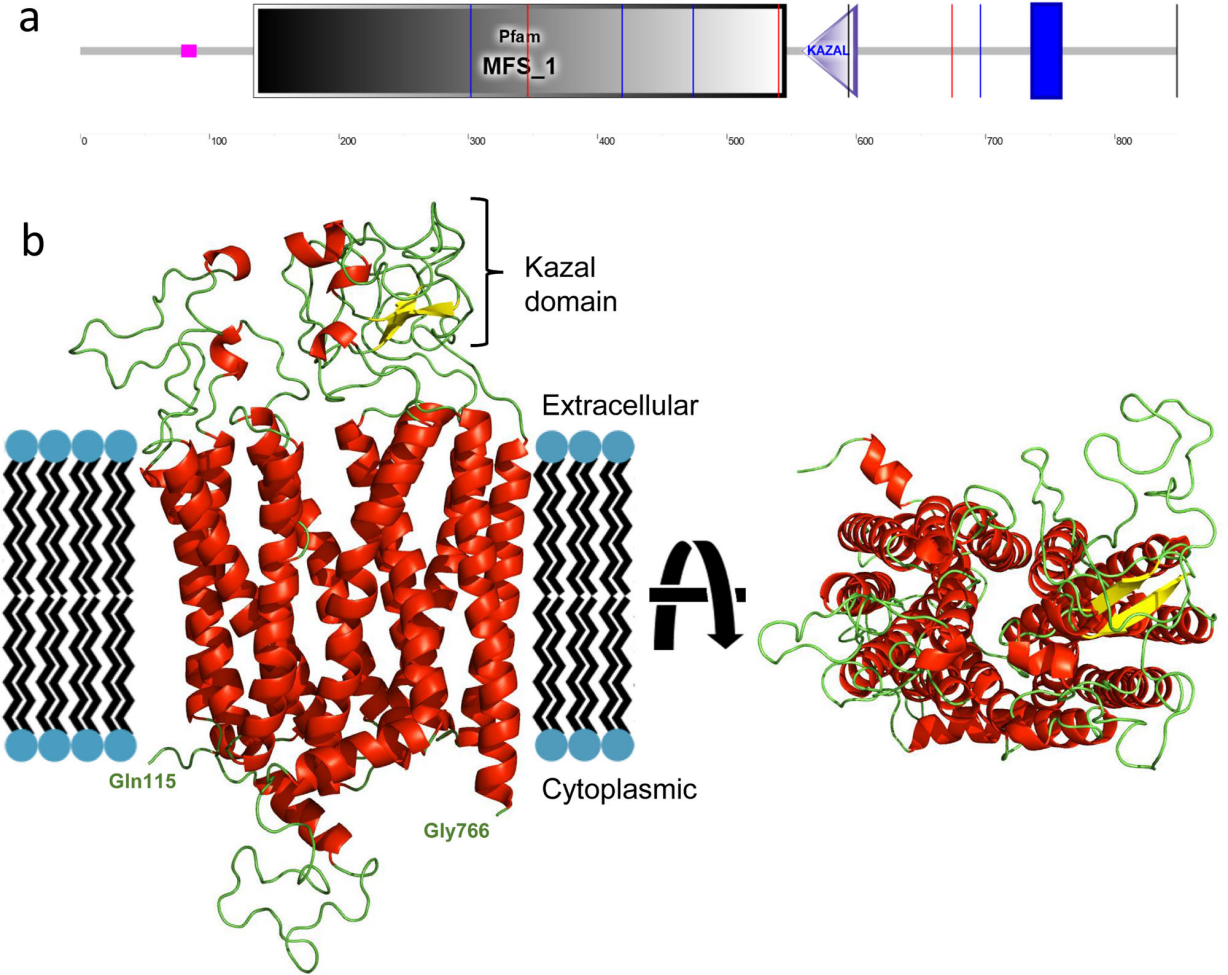
Domain architecture of human SLCO5A1. **a** Schematic representation of the protein with the indication of recognised domains. A SMART analysis to identify structural domains confirmed the presence of two modules, Major Facilitator Superfamily (MFS) and a Kazal domain, interspaced with potentially unstructured sequences. The MFS transporters are membrane proteins capable of transporting small solutes in response to chemiosmotic ion gradients^72,73^. They are represented in many organisms from *Archaea* to *Homo sapiens*. MFS proteins target a wide range of substrates, including ions, carbohydrates, lipids, amino acids and peptides, nucleosides and other small molecules and transport them in both directions across the membrane^74^. The Kazal domain is an evolutionary conserved module usually acting as a serine-protease inhibitor. **b** Predicted model of the monomeric form of SLCO5A1 from amino acids 115–766, built using the SwissModel homology server (https://swissmodel.expasy.org) and utilising the template structure pdb:7eeb. Red: alpha helices; Yellow: Beta strands; Green: Loops.

**Fig. 4:**
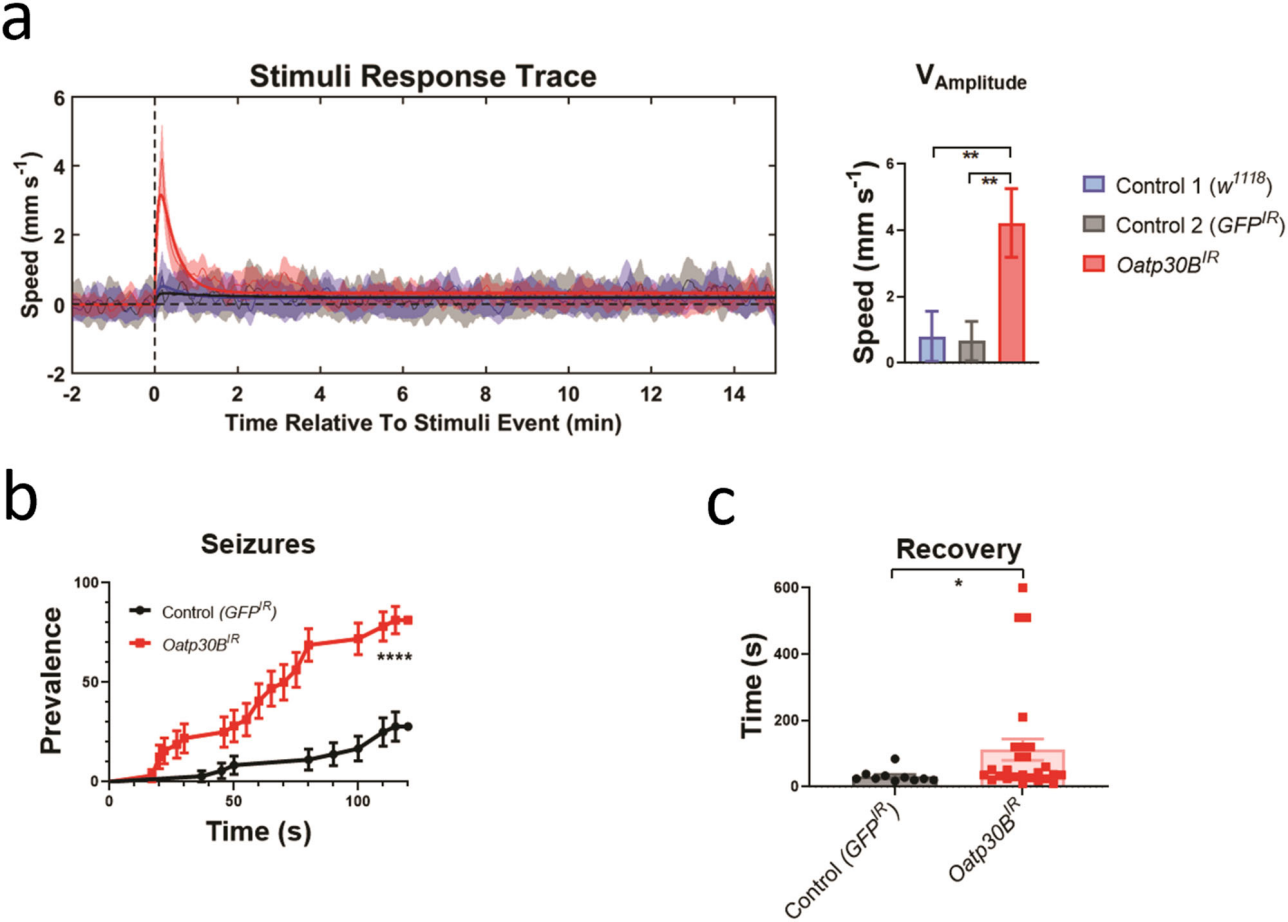
Startling reaction to trains of vibrations, increased seizure prevalence and increased post-seizure recovery time in flies with *Oatp30B* knockdown. **a** Startling reaction to trains of vibrations. The *UAS*-*Oatp30B*^*IR*^ (GD12775) transgenic or the control *UAS-GFP*^*IR*^ were driven with *nSyb-Gal4* and *Ubi-Gal80ts*. The *w*^*1118*^ strain is a control for the genetic background in absence of transgenes. Mean ±SEM **P < 0.01, One Way ANOVA, Tukey’s post-hoc test. Units are the vibration events experienced 6 times for each fly, *n* = 174–210. **b** Increased seizure prevalence. The *UAS*-*Oatp30B*^*IR*^ (GD12775) transgenic or the control *UAS-GFP*^*IR*^ were driven with *nSyb-Gal4* and *Ubi-Gal80ts*. Percent ±SE ****P < 0.0001, Log-rank (Mantel-Cox) test, *X*^2^ 24.68 for 1 df, *n* = 34–36. **c** Increased post-seizure recovery time. The *UAS*-*Oatp30B*^*IR*^ (GD12775) transgenic or the control *UAS-GFP*^*IR*^ were driven with *nSyb-Gal4* and *Ubi-Gal80ts*. Mean ±SEM *P < 0.05, Mann Whitney non-parametric test, two tails, *n* = 10–26. Only flies that displayed a seizure within 120 s as in **b** have been included in the analysis.

*SLCO5A1* is a membrane-bound organic anion transporter with no known substrate^39^ (Fig. 3). We performed a full protein BLAST (BLASTp) search of the SLCO5A1 polypeptide sequence (NP_112220.2) on *Drosophila melanogaster* to identify the closest matching sequence alignment. While several members of the Oatp family were found to have significant homology, *Oatp30B* was the family member with the highest homology and a 37.66% identity and E-value of 2 × 10^−150^ (NP_995667.1). SLCO5A1 was the closest human analogue of Oatp30B also in a reverse BLASTp. Indeed, BLASTp of Oatp30B polypeptide sequence (Q9VLB3) across all species for conserved domains reveals this gene has conserved major facilitator superfamily (MFS), OATP, and Kazal domains (Fig. 3 and Supplementary Fig. 8). We therefore used an effective RNAi transgenic line (Supplementary Fig. 9A) to assess the effect of pan-neuronal adult knockdown of *Oatp30B/SLCO5A1*. Flies with reduced *Oatp30B* levels displayed a small but significant shortening of their lifespan (Supplementary Fig. 9B) and a striking over-reaction to vibration stimuli applied through the automated Drosophila Arousal Tracking (DART) system^40^, which elicit an otherwise modest activity response in two separate control fly genotypes (Fig. 4a). Additional analysis of locomotor behaviour clarifies that *Oatp30B* knockdown did not alter the speed of flies or the duration of each activity bout or the interval in between bouts of action (Supplementary Fig. 9C–E), indicating a specific defect in excessive response to stimuli. Furthermore, *Oatp30B* knockdown led to a dramatic increase in the frequency of seizure-like events (Fig. 4b) when exposed to hyperthermia, a trigger for seizures in *Drosophila*^41^. Recovery to full motility after seizure-like events was also significantly slower in flies with *Oatp30B* knockdown (Fig. 4c). These data establish a common causal link between *Oatp30B/SLCO5A1* downregulation, startling behaviour, and susceptibility to seizure-like events.

### Gene enrichment analyses

We next sought to assess whether there was additional signal in the GWAS where sub-GWAS significant variants could inform additional contributing genes or pathways and whether there were shared genetic contributions with other psychiatric or epilepsy phenotypes. We selected all variants displaying *P* ≤ 5 × 10^−4^ and annotated these variants to the transcription start site of the nearest gene resulting in 810 unique genes. Gene enrichment analysis using one-sided hypergeometric tests^42^ identified a 12.7-fold enrichment of associated genes from the presynaptic membrane organisation gene set (five out of nine genes; gene ontology (GO):0097090) and a 14.3-fold enrichment of associated genes from the presynaptic membrane assembly gene set (five out of eight genes; GO:0097105). These genes were *PTEN, NLGN1, PTPRD, IL1RAPL1* and *NLGN4X* (Table 2). The combined PVE for the lead variants annotated to these five genes was 15.6% (25.8% with the addition of rs73293634 from the *SLCO5A1* locus and rs75042057 from the *PARD3* locus).

**Table 2:** List of top variants annotated to the five presynaptic assembly genes enriched in the European GWAS of BIS in JME (*n* = 324).

Gene	Location	Size	rsid	Beta	*P* value	PVE
*PTPRD*	chr9:8,314,246–10,613,002	2,298,757	rs1781264	1.827	1.19E-04	0.042
*NLGN1*	chr3:173,398,448–174,286,644	888,197	rs73177088	6.191	9.95E-04	0.044
*NLGN4X*	chrX:5,890,042–6,228,867	338,826	rs146813567	−2.898	3.06E-04	0.039
*IL1RAPL1*	chrX:28,587,446–29,956,718	1,369,273	rs5943492	1.039	8.73E-04	0.043
*PTEN*	chr10:87,862,563–87,971,930	109,368	rs112050451	5.158	1.27E-03	0.041

Variants with *P* ≤ 5 × 10^−4^ were annotated to the gene with the nearest transcription start site using the Ensembl Variant Effect Predictor (v94)^62^. This gene set was used as input in a GO enrichment analysis^63,64^, to test for enrichment in annotated pathways. One-sided hypergeometric tests were completed to identify over-representation of pathways^42^. To reduce the risk of false positive results, a permutation procedure^65^ was employed by randomly shuffling GWAS *p* values 2000 times, each time re-applying the *P* ≤ 5 × 10^−4^ threshold and calculating the hypergeometric test statistics. For each pathway, the final permutation-based *p* value was calculated as the percentage of the 2000 permutations that produced a *p* value less than or equal to the *p* value calculated from the non-permuted data. A pseudo count was added during this calculation to prevent calculating *p* values equal to 0.

*PTPRD*, Protein Tyrosine Phosphatase Receptor Type D; *NLGN1*, Neuroligin 1; *NLGN4X*, Neuroligin 4 X-Linked; *IL1RAPL1*, Interleukin 1 Receptor Accessory Protein Like 1; *PTEN*, Phosphatase and Tensin Homologue.

The permutation tests of presynaptic membrane organisation (GO:0097090) over-enrichment and of presynaptic membrane assembly (GO:0097104) over-enrichment both produced permutation-based *p* values of 0.0005.

Investigation of these 810 genes revealed further^43–45^ that there was a significant overlap with genes reported in the GWAS catalogue that contribute to phenotypes relevant to the predominance of JME seizures on awakening, impulsivity and metabolism: chronotype (66 out of 522 genes overlap, *P* = 2.92 × 10^−12^), obesity-related traits (77 out of 662 overlap, *P* = 2.69 × 10^−12^), general risk tolerance (30 out of 238 overlap, *P* = 2.30 × 10^−5^), and adventurousness (21/134, *P* = 3.70 × 10^−5^).

### Polygenic risk score analysis

Given impulsivity is a major component of ADHD, risk taking, bipolar disorder and epilepsy, we tested and found that a higher ADHD polygenic risk score (PRS) was significantly associated with a higher BIS-Brief score (*p* = 1.60 × 10^−3^) (Supplementary Fig. 10). It should be noted that the lead *SLCO5A1* SNP, rs73293634, was not present in the ADHD GWAS from which the PRS was calculated, but rs146866040 which is in high LD did not show evidence of association itself with ADHD (OR (SE) = 0.9481(0.0562), *p* = 0.34)^46^. The rs75042057 SNP on Chr10 was also not present in the ADHD dataset nor was there a proxy with *R*^2^ > 0.6 available. The risk-taking PRS was also nominally associated with a higher BIS-Brief score (*p* = 0.018). PRSs for bipolar disorder, generalised and focal epilepsy did not reach statistical significance for association with BIS-Brief score at the 5% or Bonferroni-corrected level of 1% (*P* = 0.08, 0.33 and 0.96, respectively) (Supplementary Table 4). Altogether this suggests that the impulsive trait seen in JME is an endophenotype that shares genetic architecture with impulsivity in the general population as well as with individuals diagnosed with ADHD.

## Discussion

This is a GWAS of trait impulsivity in a neuropsychiatric disorder and we present convergent evidence for the role of SLCO5A1 in impulsivity and seizure susceptibility through triangulation^47^ with GWAS, independent replication, colocalization with gene expression and functional evaluation in *Drosophila*^48^. While several Oatp family members display significant homology to SLCO5A1, the identified Oatp30B was the closest polypeptide in a BLASTp search and SLCO5A1 was the human polypeptide with the highest homology in a reverse BLASTp search. Therefore, whereas our analysis does not rule out some contribution by other closely related Oatp genes, for instance *Oatp26F*, it has identified a major role of *Oatp30B* in regulating startling and seizure-like behaviour in *Drosophila*. In contrast to human *SLCO5A1*, *Oatp30B* is expressed in the nervous system at constant low to moderate levels throughout fly stages, from development to adulthood. This enables investigation of gene function in vivo, in adult flies, although it limits generalisation as an *SLCO5A1*-linked disease model.

One GWAS of impulsive traits in the general population identified genome-wide significant association with variants in the *CADM2* gene. *CADM2* encodes a cell adhesion protein from the SynCam Immunoglobulin superfamily of recognition molecules, important for synaptic organisation and specificity; association of variants at the *CACNA1I* locus has been observed in previous studies with schizophrenia^9^. Our GWAS did not show significant association with these previously reported general population-associated variants at the *CADM2* and *CACNA1I* loci^9^ (*P* = 0.152, *beta* = −0.52 for rs139528938; and *P* = 0.32, *beta* = −0.35 for rs4522708; the latter a SNP with *r*^2^ = 0.87 with the reported SNP, rs199694726, in our BIS-Brief dataset). Genome-wide summary statistics were not available to make additional comparisons. Genome-wide summary statistics were available for the risk-taking phenotype in the UK Biobank^31^, in which we observed replication of our lead genome-wide significant *SLCO5A1* variant, rs73293634.

Previous expression studies show that *SLCO5A1* upregulates gene sets implicated in cell adhesion, synapse assembly and organisation, principally belonging to the cadherin superfamily^39^; and the enrichment for presynaptic membrane assembly and organisation pathways in our dataset includes genes encoding trans-synaptically interacting proteins that are implicated in a wide range of neuropsychiatric disorders^49,50^. Genetic correlation between ADHD and the BIS-Brief score suggests converging genetic influences across ADHD and epilepsy. Taken together, these results support an important role for specific cell recognition molecules in the organisation of synaptic connections as a mechanism for variation in impulsivity across health and disease^51^.

While prefrontal-striatal inhibitory control networks are implicated in impulse control, specifically between mPFC and nucleus accumbens^18,20^, a role for these limbic networks has only been hinted at in epilepsy. Striato-nigral circuits, preferentially involving the ventral striatum, have long ago been implicated in the *regulation* of generalised seizures in rodent models of generalised epilepsy^19^. Recently, an *initiating* role for cortico-striatal networks in absence seizures with generalised spike-and-wave discharges has been shown in the mouse model of genetic epilepsy caused by haploinsufficiency of *STXBP1*^52^, specifically by reduced cortical excitatory transmission onto striatal fast-spiking interneurons. The startling and the seizure-like phenotype of the *SLCO5A1/Oatp30B* knockdown in *Drosophila* suggests the genetic co-causality of startling and seizures. While it is not possible to define startling as the *Drosophila* equivalent of impulsivity, the two traits share some commonality in the lack of moderation in behaviour. This offers some additional support to the idea that excitatory-inhibitory imbalance in the prefrontal-striatal network may predispose simultaneously to epilepsy and impulsivity substrates and invites new approaches to neuromodulation of generalised seizures.

## Methods

### Human participants

We collected cross-sectional clinical and genetic data from the Biology of Juvenile Myoclonic Epilepsy (BIOJUME) consortium study, which focuses on gathering cases with JME (*n* = 864)^25^. Inclusion criteria have been discussed previously^6^. BIOJUME is a study across 50 sites in 10 countries (Appendix). Furthermore, all participants’ medical history was reviewed by a phenotyping committee to validate the diagnosis of JME. Written informed consent was obtained from all participants prior to inclusion in the study and ethical approval from the UK Health Research Authority, South Central Oxford C Research Ethics Committee (16/SC/0266) and all other collaborating sites was obtained. The SickKids Research Ethics Board of The Hospital for Sick Children (1000033784) also gave ethical approval for this work.

### Barratt impulsivity scale-brief (BIS-Brief)

We collected self-rating of trait impulsivity through the BIS-brief^6,30^. The BIS-Brief is a short version of the BIS, one of the most commonly used measures of impulsiveness. The current version of BIS (BIS-11) includes 30-items measuring 3 theoretical subtraits: attentional, motor, and non-planning impulsiveness. BIS-Brief is a unidimensional scale including 8 of the original BIS-11 items generating a total score ranging from 8 to 32. BIS-Brief demonstrated similar indices of construct validity observed for the BIS-11 total score. Using BIS-Brief in large epidemiological studies of psychiatric disorders reduces the burden on respondents without loss of information^29^.

### Genotyping quality control

DNA was extracted from blood by each consortium site and sent to The Centre for Applied Genomics at The Hospital for Sick Children in Toronto for genotyping. We genotyped participants’ DNA in four batches (*n* = 702) using the Illumina Omni 2.5 array. SNPs were called using the self-clustering method in Genome Studio. We performed quality control (QC) for each genotyped batch using PLINK v1.90b6.18^53^ and custom in-house scripts. Briefly, we removed individuals and variants with call rates below 90%; samples with sex mismatches and/or high heterozygosity; males with heterozygous calls for X chromosome markers (non-pseudoautosomal region); and females with non-missing calls for markers on the Y chromosome. We retained heterozygous calls for mitochondrial markers in both sexes (i.e., due to heteroplasmy). We obtained an unrelated sample by using KING v.2.2.4 software’s^54^ --unrelated option (that is, those with estimated kinship coefficient <0.088). We corrected and updated the ped file with all found relationships, and identified markers with Mendelian errors using PEDSTATS 0.6.12^55^. We flagged 399 markers but did not remove those out of Hardy–Weinberg Equilibrium (*P* < 10^−4^). We conducted principal component analysis adjusted using the kinship matrix output by KING using PC-AiR in the GENESIS v2.16.0 package^56^.

We performed quality control on each genotyping batch separately, followed by removal of ambiguous A/T, G/C SNPs, chr0 SNPs, indels, monomorphic variants, and duplicate variants; and performed strand alignment using Will Rayner’s alignment files (www.well.ox.ac.uk/~wrayner/strand/), then merged all batches. We re-analysed and removed cryptic relationships across batches. The final merged set contained 1,489,917 variants, 695 individuals (241 males, 454 females) including 23 related pairs (for association analyses however, an unrelated set was selected).

### Genotype imputation

We used the McCarthy Tools v4.3.0 to prepare the genotype data for imputation (www.well.ox.ac.uk/~wrayner/tools/HRC-1000G-check-bim-v4.3.0.zip) using TOPMed as the reference panel (r2@1.0.0) on the TOPMed imputation server^57–59^. We converted coordinates from hg37 to hg38 coordinates using strand files (www.well.ox.ac.uk/~wrayner/strand/InfiniumOmni2-5-8v1-4_A1-b38-strand.zip). We merged the pseudoautosomal region (PAR) using PLINK’s --merge-x option and checked variants using the HRC checking tool. We removed a total of 282,660 variants due to no matches in the reference (but still analysed for association with BIS-Brief afterwards), and 1,739,329 variants remained for imputation on the server. We used Eagle v2.4 for phasing, and minimac v4 v1.0.2 for imputation. We kept variants with imputation quality score *r*^2^ > 0.4 and MAF > 1% for analysis. A total of 8,950,360 variants remained for association analysis.

### Genome-wide association analysis

We included for analysis 381 individuals who passed phenotype QC with complete BIS-Brief rating. From these, four failed genotyping QC, and one individual was removed due to cryptic relatedness (*n* = 376). The mega-GWAS analysis consisted of a total of 372 unrelated individuals adjusted for sex, genotyping batch, and population stratification (Supplementary Fig. 1). The mega-GWAS was used for colocalization analysis of the genome-wide association peak on chromosome 8. We identified 329 patients as European ancestry (defined as within 6 standard deviations from the 1000 Genomes^60^ European cluster in a principal component analysis). Among these, five patients had missing information on seizure frequency, so we used 324 individuals for the genome-wide association analysis. The current sample size is sufficient to detect genetic variants that explain 12% of the variance in the BIS-Brief score with 80% power after adjusting for multiple hypothesis testing at the genome-wide significance level. We adjusted for sex, genotyping batch, age at consent, population stratification, and the frequency of myoclonus or absence seizures. The relationship of the frequency of myoclonus or absence seizures, and its relationship with ASM and sex with trait impulsivity in JME, has been described previously and was thus adjusted for in current regression analyses^6,25^. All analyses were conducted in the European subset unless noted otherwise. Chromosome X (non-pseudoautosomal region) was analysed with males coded as zero for the reference allele and two for the alternate allele, under the assumption of X-inactivation^61^. Genome-wide significant loci were further investigated for replication of association with risk-taking phenotypes in the general population using publicly available summary statistics^31,32^.

### Gene enrichment analysis

Variants with *P* ≤ 5 × 10^−4^ were annotated to the gene with the nearest transcription start site using the Ensembl Variant Effect Predictor (v94)^62^. This gene set was used as input in a GO enrichment analysis^63,64^, to test for enrichment in annotated pathways. One-sided hypergeometric tests were completed to identify over-representation of pathways^42^. To reduce the risk of false positive results, a permutation procedure^65^ was employed by randomly shuffling GWAS *p* values 2000 times, each time re-applying the *P* ≤ 5 × 10^−4^ threshold and calculating the hypergeometric test statistics. For one pathway, the final permutation-based *p* value was calculated as the percentage of the 2000 permutations that produced a *p* value less than or equal to the *p* value calculated from the non-permuted data. A pseudo count was added during this calculation to prevent calculating *p* values equal to 0.

### Phenome-wide association study (PheWAS) analysis

We queried the top associated genome-wide variant and the top associated variant for each of the nine presynaptic assembly enriched genes across PheWAS databases: GWAS Atlas (https://atlas.ctglab.nl/), Global Biobank Engine^66^, PheWeb^67^, and Gene Atlas^68^.

We used PheWeb portals:

- UK Biobank: https://pheweb.org/MGI-freeze2/
- Oxford Brain Imaging Genetics (BIG) Project: http://big.stats.ox.ac.uk/
- fastGWA: https://yanglab.westlake.edu.cn/resources/ukb_fastgwa/imp/
- https://pheweb.org/UKB-SAIGE/

### PRS analysis

Clumping and thresholding were used to calculate ADHD, risk taking, bipolar disorder, generalised epilepsy, and focal epilepsy PRS in individuals of European ancestry using PLINK v1.9^53^. Five PRS were calculated. A Bonferroni-corrected critical value for significance would therefore be *p* < 0.05/5 = 0.01. The source of summary statistics used, variant filtering, clumping and thresholding details are summarised in Supplementary Table 1. PRS values were generated by weighting selected SNPs after clumping and thresholding by the additive scale effect (log_10_(OR)/Beta), and then summing over the variants. The PRS values were then centred to the mean. Association of PRSs with BIS-Brief was tested using linear regression with age, sex, and frequency of absence/myoclonic seizure as covariates in the model.

### Colocalization analysis

We used the Simple Sum 2^36^ and COLOC2^69^ colocalization methods as implemented in LocusFocus^70^ (v1.4.9) to test for colocalization of the genome-wide peaks with eQTL analyses in brain tissues in GTEx v8^12^, PsychENCODE^34^, and fetal brain^35^. For the genome-wide associated locus on chromosome 8, we performed colocalization analysis using both the mega-GWAS and Europeans-only GWAS. The required significance threshold, after multiple testing of all colocalization datasets analysed was 0.01.

### Domain architecture of SLCO5A1

A BLAST search against the entire Protein Data Base (PDB) identified only one hit with a convincingly high E-value (1e-55) that pointed to the Chain L of the Kazal-like domain containing mice protein (7EEB). The search had a 26% identity and a coverage of 74%. After this hit, the other four identified sequences had *E* values > 0.002, clearly distinguishing between significant and non-significant hits. 7EEB is a large complex containing several subunits, among which is *SLCO6C1*, which is the region scoring for *SLCO5A1*.

### Phenotypic variance explained

To assess the PVE by a SNP or a group of SNPs, we calculated the partial *r*2 as the proportion of the residual sum of squares (RSS) reduced when adding the SNP (or group of SNPs) to the base regression model with all covariates.

### siRNA probe design and knockdown of *Oatp30B* in *Drosophila melanogaster*

### Drosophila

Flies were maintained and crossed at 18 °C. All ageing was done in a controlled environment of 29 °C and 60% humidity.

### Stocks

*ubiGal80*^*ts*^ // *UAS*-*Oatp30B*^*IR*^ (GD12775) obtained from the VDRC // *w*^*1118*^, *nSyb-Gal4, TubGal4* and *UAS-GFP*^*IR*^ obtained from the BDSC.

### Lifespan

Lifespan analysis was performed as previously reported^41^. All crosses were maintained at 18 °C during the developmental stages of the progeny. Newly eclosed adult flies were collected within 5 days at 18 °C. Females and males were pooled together and equally distributed within vials.

### Motor behaviour assay

Single-fly tracking was carried out as previously described^41^. In each of three experiments, up to 12 flies per genotype, aged 15 days (adult stage) at 29 °C to allow RNAi expression and knockdown, were placed into individual round six-wells arenas. The protocol used consisted of 6 stimuli events equally split during a period of 2 h and 15 min, the first one starting after 30 min of recording, and the last one 30 min before the end of the protocol. Each stimuli event was composed of five vibrations of 200 ms spaced by 500 ms. The x/y position of each single fly was tracked and analysed using the DART software in order to evaluate the relative speed and activity before, during and after the stimuli event. The speed analysis was used for the “Stimuli Response Trace” and the general activity used to deduce “Active Speed”, “Mean Bout Length” and “Inter-Bout Interval”, using a custom-made modification of the DART software^40^.

### Heat-induced seizure assay

Flies aged 15 days at 29 °C to allow RNAi expression and knockdown were isolated into new plastic vials without food for 10–20 min before immersion in a 42 °C water bath for 120 seconds. Each tube was video recorded during and post immersion and seizures were defined as a period of brief leg twitches, convulsions, and failure to maintain standing posture. Flies were, thereafter, allowed to recover at room temperature and the time to recover from seizure was calculated only for flies that had undergone seizures. All experiments were randomised and double-blinded.

### RNA extraction and qPCR

RNA was extracted as previously reported^71^ from 15 adult flies of both sexes, aged 15 days at 29 °C to allow RNAi expression and knockdown, using TriZol (Thermo-Fischer). cDNA was generated using SuperScript III Reverse Transcriptase (Thermo-Fischer). Quantitative PCR was performed in combination with qPCRBIO SyGreen Blue mix (PCR Biosystems) on Quantstudio 7 from real-time PCR system (Thermo-Fischer). *eIF4a* was used as housekeeping control. The following oligos were used: *Oatp30B* Fw (GAATCCGACCAACCGCCTGA), *Oatp30B* Rv (ATGGATTCCTGCCGCCTGTG), *eIF4a* Fw (CGTGAAGCAGGAGAACTGG), *eIF4a* Rv (CATCTCCTGGGTCAGTTG).

### Reporting summary

Further information on research design is available in the Nature Research Reporting Summary linked to this article.

### Supplementary information

Supplementary Material

Reporting Summary

## Data Availability

eQTL data are available for download from GTEx (https://gtexportal.org/home), PsychENCODE (http://resource.psychencode.org/), and fetal brains (10.6084/m9.figshare.6881825). GWAS summary statistics for this study are available for download from our website (https://lab.research.sickkids.ca/strug/softwareandresources/).

## References

[1] Daruna, J. H. & Barnes, P. A. *In:* The *im*pulsiv*e* client: theory, research and treatment (eds W. G. McCown, J. L. Johnson, & M. B. Shure) 23–37 (American Psychological Association, 1993).

[2] Niv S, Tuvblad C, Raine A, Wang P, Baker LA (2012). Heritability and longitudinal stability of impulsivity in adolescence. Behav. Genet..

[3] Dalley JW, Robbins TW (2017). Fractionating impulsivity: neuropsychiatric implications. Nat. Rev. Neurosci..

[4] Ramirez-Martin A, Ramos-Martin J, Mayoral-Cleries F, Moreno-Kustner B, Guzman-Parra J (2020). Impulsivity, decision-making and risk-taking behaviour in bipolar disorder: a systematic review and meta-analysis. Psychol. Med..

[5] Smith A, Syvertsen M, Pal DK (2020). Meta-analysis of response inhibition in juvenile myoclonic epilepsy. Epilepsy Behav..

[6] Shakeshaft, A. et al. Trait impulsivity in juvenile myoclonic epilepsy. *Ann Clin Transl Neurol*, 10.1002/acn3.51255 (2020). 10.1002/acn3.51255 PMC7818143 33264519

[7] Wandschneider B (2013). Risk-taking behavior in juvenile myoclonic epilepsy. Epilepsia.

[8] Malloy-Diniz L, Fuentes D, Leite WB, Correa H, Bechara A (2007). Impulsive behavior in adults with attention deficit/ hyperactivity disorder: characterization of attentional, motor and cognitive impulsiveness. J. Int. Neuropsychol. Soc..

[9] Sanchez-Roige S (2019). Genome-wide association studies of impulsive personality traits (BIS-11 and UPPS-P) and drug experimentation in up to 22,861 adult research participants identify loci in the CACNA1I and CADM2 genes. J. Neurosci..

[10] Barbeira AN (2018). Exploring the phenotypic consequences of tissue specific gene expression variation inferred from GWAS summary statistics. Nat. Commun..

[11] Schizophrenia Working Group of the Psychiatric Genomics, C. (2014). Biological insights from 108 schizophrenia-associated genetic loci. Nature.

[12] GTEx Consortium. (2013). The genotype-tissue expression (GTEx) project. Nat. Genet..

[13] O’Muircheartaigh J (2011). Focal structural changes and cognitive dysfunction in juvenile myoclonic epilepsy. Neurology.

[14] Keller SS (2011). Microstructural and volumetric abnormalities of the putamen in juvenile myoclonic epilepsy. Epilepsia.

[15] Landvogt C, Buchholz H-G, Bernedo V, Schreckenberger M, Werhahn KJ (2010). Alteration of dopamine D2/D3 receptor binding in patients with juvenile myoclonic epilepsy: alteration of dopamine D2/D3 receptor binding in JME. Epilepsia.

[16] Ciumas C (2008). Reduced dopamine transporter binding in patients with juvenile myoclonic epilepsy. Neurology.

[17] Dalley JW, Everitt BJ, Robbins TW (2011). Impulsivity, compulsivity, and top-down cognitive control. Neuron.

[18] Dalley JW, Roiser JP (2012). Dopamine, serotonin and impulsivity. Neuroscience.

[19] Deransart C, Vercueil L, Marescaux C, Depaulis A (1998). The role of basal ganglia in the control of generalized absence seizures. Epilepsy Res..

[20] Cho SS (2013). Morphometric correlation of impulsivity in medial prefrontal cortex. Brain Topogr..

[21] Santos BPD (2017). Genetic susceptibility in juvenile myoclonic epilepsy: systematic review of genetic association studies. PLoS One.

[22] International League Against Epilepsy Consortium on Complex Epilepsies. (2018). Genome-wide mega-analysis identifies 16 loci and highlights diverse biological mechanisms in the common epilepsies. Nat. Commun..

[23] Bai D (2008). DNA variants in coding region of EFHC1: SNPs do not associate with juvenile myoclonic epilepsy. Epilepsia.

[24] Bailey JN (2018). Variant intestinal-cell kinase in juvenile myoclonic epilepsy. N. Engl. J. Med..

[25] Shakeshaft A (2022). Sex-specific disease modifiers in juvenile myoclonic epilepsy. Sci. Rep..

[26] Li M (2021). Antisense oligonucleotide therapy reduces seizures and extends life span in an SCN2A gain-of-function epilepsy model. J. Clin. Invest..

[27] Karalis V, Bateup HS (2021). Current approaches and future directions for the treatment of mTORopathies. Dev. Neurosci..

[28] Hall M-H, Smoller JW (2010). A new role for endophenotypes in the GWAS era: functional characterization of risk variants. Harv. Rev. Psychiatry..

[29] Manchia M (2013). The impact of phenotypic and genetic heterogeneity on results of genome wide association studies of complex diseases. PLoS One.

[30] Steinberg L, Sharp C, Stanford MS, Tharp AT (2013). New tricks for an old measure: the development of the Barratt Impulsiveness Scale-Brief (BIS-Brief). Psychol. Assess..

[31] Watanabe K (2019). A global overview of pleiotropy and genetic architecture in complex traits. Nat. Genet..

[32] *Neale’s* Lab UK Biobank GWAS Results Round 2 (Imputed v3 - File Manifest Release 20180731), http://www.nealelab.is/uk-biobank.

[33] Kinirons P (2008). A novel locus for idiopathic generalized epilepsy in French-Canadian families maps to 10p11. Am. J. Med. Genet. A.

[34] Wang, D. et al. Comprehensive functional genomic resource and integrative model for the human brain. *Science* **362**, 10.1126/science.aat8464 (2018). 10.1126/science.aat8464 PMC6413328 30545857

[35] O’Brien HE (2018). Expression quantitative trait loci in the developing human brain and their enrichment in neuropsychiatric disorders. Genome Biol..

[36] Wang F, Panjwani N, Wang C, Sun L, Strug LJ (2022). A flexible summary statistics-based colocalization method with application to the mucin cystic fibrosis lung disease modifier locus. Am. J. Hum. Genet..

[37] BrainSpan Atlas of the Developing Human Brain [Internet], http://brainspan.org.

[38] Sunkin SM (2013). Allen Brain Atlas: an integrated spatio-temporal portal for exploring the central nervous system. Nucleic Acids Res..

[39] Sebastian K (2013). Characterization of SLCO5A1/OATP5A1, a solute carrier transport protein with non-classical function. PLoS One.

[40] Faville R, Kottler B, Goodhill GJ, Shaw PJ, van Swinderen B (2015). How deeply does your mutant sleep? Probing arousal to better understand sleep defects in Drosophila. Sci. Rep..

[41] Mazaud D (2019). Transcriptional regulation of the glutamate/GABA/glutamine cycle in adult glia controls motor activity and seizures in drosophila. J. Neurosci..

[42] Falcon S, Gentleman R (2007). Using GOstats to test gene lists for GO term association. Bioinformatics.

[43] Liberzon A (2015). The Molecular Signatures Database (MSigDB) hallmark gene set collection. Cell Syst..

[44] Meissner A (2008). Genome-scale DNA methylation maps of pluripotent and differentiated cells. Nature.

[45] Mikkelsen TS (2007). Genome-wide maps of chromatin state in pluripotent and lineage-committed cells. Nature.

[46] Demontis D (2019). Discovery of the first genome-wide significant risk loci for attention deficit/hyperactivity disorder. Nat. Genet..

[47] Lawlor DA, Tilling K, Davey Smith G (2016). Triangulation in aetiological epidemiology. Int. J. Epidemiol..

[48] Parker L, Howlett IC, Rusan ZM, Tanouye MA (2011). Seizure and epilepsy: studies of seizure disorders in Drosophila. Int. Rev. Neurobiol..

[49] Uhl GR, Martinez MJ (2019). PTPRD: neurobiology, genetics, and initial pharmacology of a pleiotropic contributor to brain phenotypes. Ann. N Y Acad. Sci..

[50] Hu Z, Xiao X, Zhang Z, Li M (2019). Genetic insights and neurobiological implications from NRXN1 in neuropsychiatric disorders. Mol. Psychiatry.

[51] Sanes JR, Zipursky SL (2020). Synaptic specificity, recognition molecules, and assembly of neural circuits. Cell.

[52] Miyamoto H (2019). Impaired cortico-striatal excitatory transmission triggers epilepsy. Nat. Commun..

[53] Purcell S (2007). PLINK: a tool set for whole-genome association and population-based linkage analyses. Am. J. Hum. Genet..

[54] Manichaikul A (2010). Robust relationship inference in genome-wide association studies. Bioinformatics.

[55] Wigginton JE, Abecasis GR (2005). PEDSTATS: descriptive statistics, graphics and quality assessment for gene mapping data. Bioinformatics.

[56] Conomos MP, Miller MB, Thornton TA (2015). Robust inference of population structure for ancestry prediction and correction of stratification in the presence of relatedness. Genet. Epidemiol..

[57] Das S (2016). Next-generation genotype imputation service and methods. Nat. Genet..

[58] Fuchsberger C, Abecasis GR, Hinds DA (2015). minimac2: faster genotype imputation. Bioinformatics.

[59] Taliun D (2021). Sequencing of 53,831 diverse genomes from the NHLBI TOPMed Program. Nature.

[60] Genomes Project Consortium (2015). A global reference for human genetic variation. Nature.

[61] Chen B, Craiu RV, Strug LJ, Sun L (2021). The X factor: a robust and powerful approach to X-chromosome-inclusive whole-genome association studies. Genet. Epidemiol..

[62] McLaren W (2016). The ensembl variant effect predictor. Genome Biol..

[63] Ashburner M (2000). Gene ontology: tool for the unification of biology. The Gene Ontology Consortium. Nat. Genet..

[64] Gene Ontology, C. (2021). The Gene Ontology resource: enriching a GOld mine. Nucleic Acids Res..

[65] Backes C (2014). Systematic permutation testing in GWAS pathway analyses: identification of genetic networks in dilated cardiomyopathy and ulcerative colitis. BMC Genomics.

[66] McInnes G (2019). Global Biobank Engine: enabling genotype-phenotype browsing for biobank summary statistics. Bioinformatics.

[67] Gagliano Taliun SA (2020). Exploring and visualizing large-scale genetic associations by using PheWeb. Nat. Genet..

[68] Canela-Xandri O, Rawlik K, Tenesa A (2018). An atlas of genetic associations in UK Biobank. Nat. Genet..

[69] Dobbyn A (2018). Landscape of conditional eQTL in dorsolateral prefrontal cortex and co-localization with schizophrenia GWAS. Am. J. Hum. Genet..

[70] Panjwani N (2020). LocusFocus: web-based colocalization for the annotation and functional follow-up of GWAS. PLoS Comput. Biol..

[71] Napoletano F (2011). Polyglutamine Atrophin provokes neurodegeneration in Drosophila by repressing fat. EMBO J..

[72] Pao SS, Paulsen IT, Saier MH (1998). Major facilitator superfamily. Microbiol. Mol. Biol. Rev..

[73] Walmsley AR, Barrett MP, Bringaud F, Gould GW (1998). Sugar transporters from bacteria, parasites and mammals: structure-activity relationships. Trends Biochem. Sci..

[74] Madej MG, Dang S, Yan N, Kaback HR (2013). Evolutionary mix-and-match with MFS transporters. Proc. Natl Acad. Sci. USA.

