# Supplementary material for: SLCO5A1 and synaptic assembly genes contribute to impulsivity in juvenile myoclonic epilepsy
Source: NPJ Genom Med. 2023 Sep 28;8:28. doi: 10.1038/s41525-023-00370-z (PMC10539321; doi:10.1038/s41525-023-00370-z)
Supplement: Supplementary file 1 — Supplementary Material [file 41525_2023_370_MOESM1_ESM.pdf]

Supplementary Figures

Supplementary Figure 1. Flowchart of inclusion for the mega-GWAS and the European GWAS

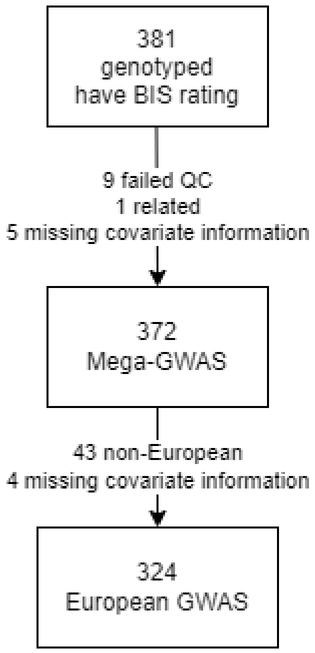

The mega-GWAS includes individuals of all ethnic backgrounds, while the main European GWAS includes those individuals within six standard deviations from the 1000 Genomes-defined European cluster.

Supplementary Figure 2. The distribution of BIS-Brief

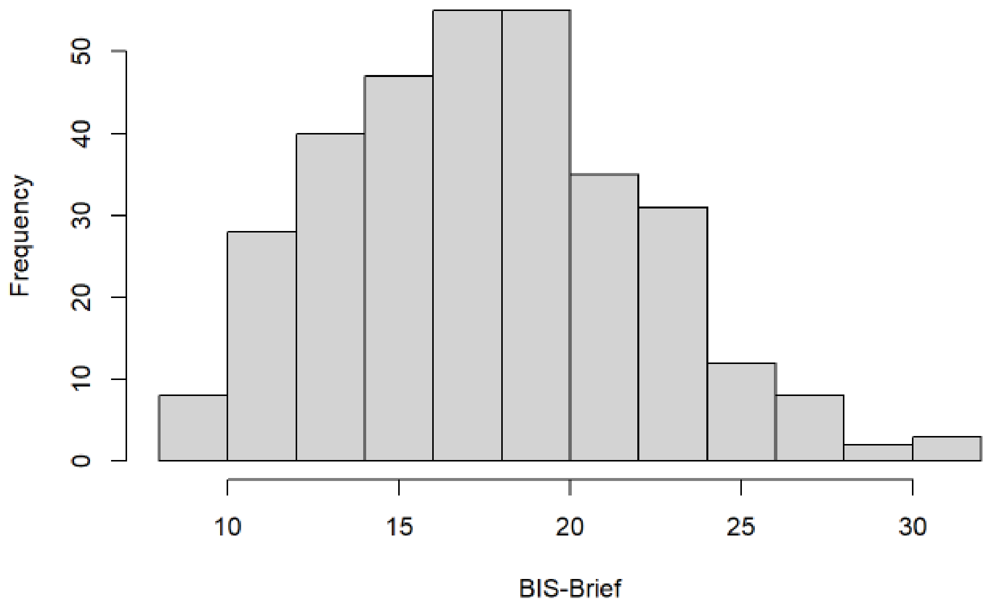

The plot shows the distribution of BIS-Brief in 324 European subjects with JME.

Supplementary Figure 3. QQ plot of BIS-Brief GWAS (GC lambda = 0.998)

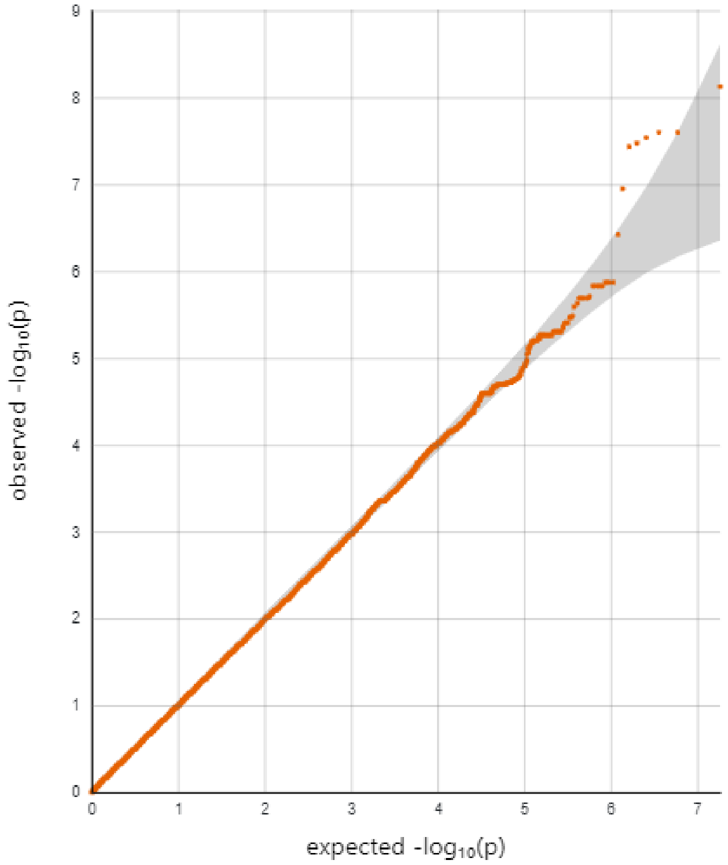

Linear regression was used to test association of each SNP with BIS-Brief. Sex, genotyping batch, age at consent, first 3 PCs, and the frequency of myoclonus or absence seizures were included as covariates in the model.

Supplementary Figure 4. Histogram of p-values for BIS-Brief GWAS

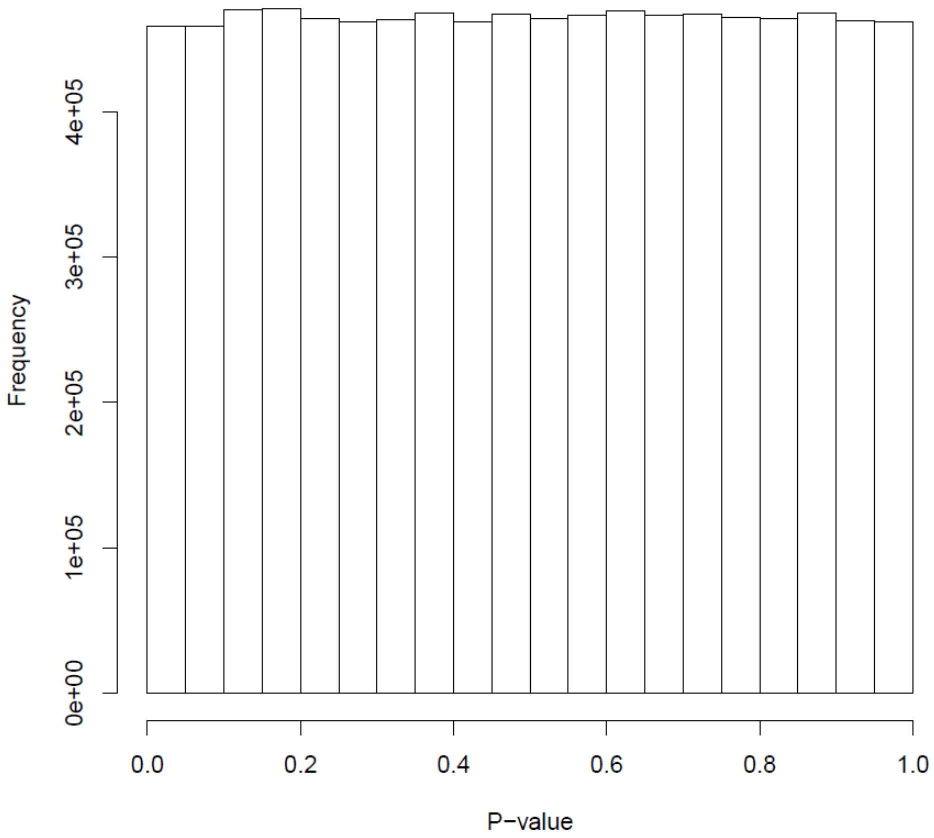

Linear regression was used to test association of each SNP with BIS-Brief. Sex, genotyping batch, age at consent, first 3 PCs, and the frequency of myoclonus or absence seizures were included as covariates in the model.

Supplementary Figure 5. Distribution of BIS-Brief by rs73293634 and rs75042057 genotypes

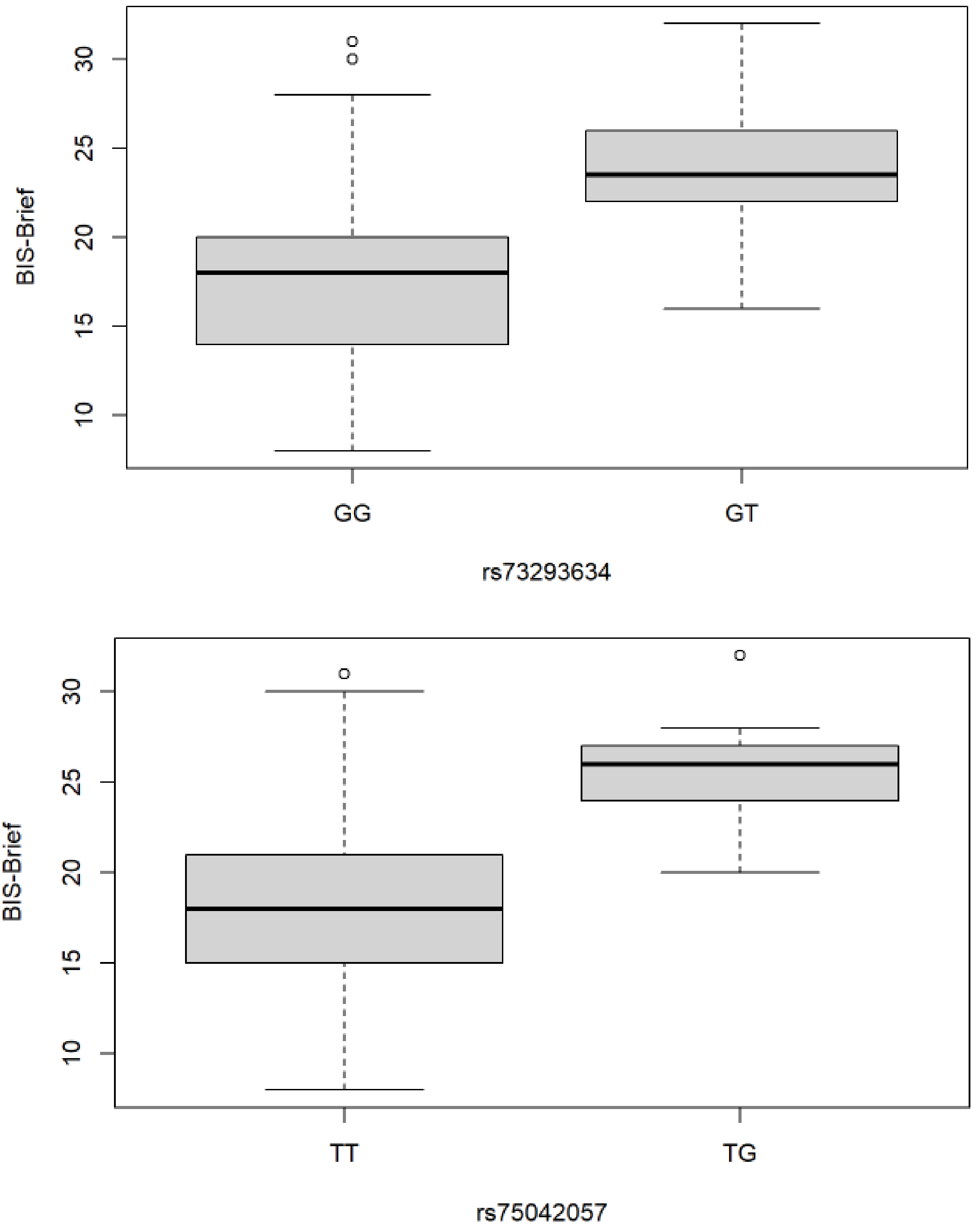

The center lines represent the 50th percentile (median) and the bounds of the boxes are the 75th and 25th percentiles (interquartile range) with the whiskers being the largest value within 1.5 times the interquartile range above the 75th percentile and smallest values within 1.5 times the interquartile range below the 25th percentile.

Supplementary Figure 6. Principal component analysis (PCA) of all study samples with the 1000 Genomes (phase 3)

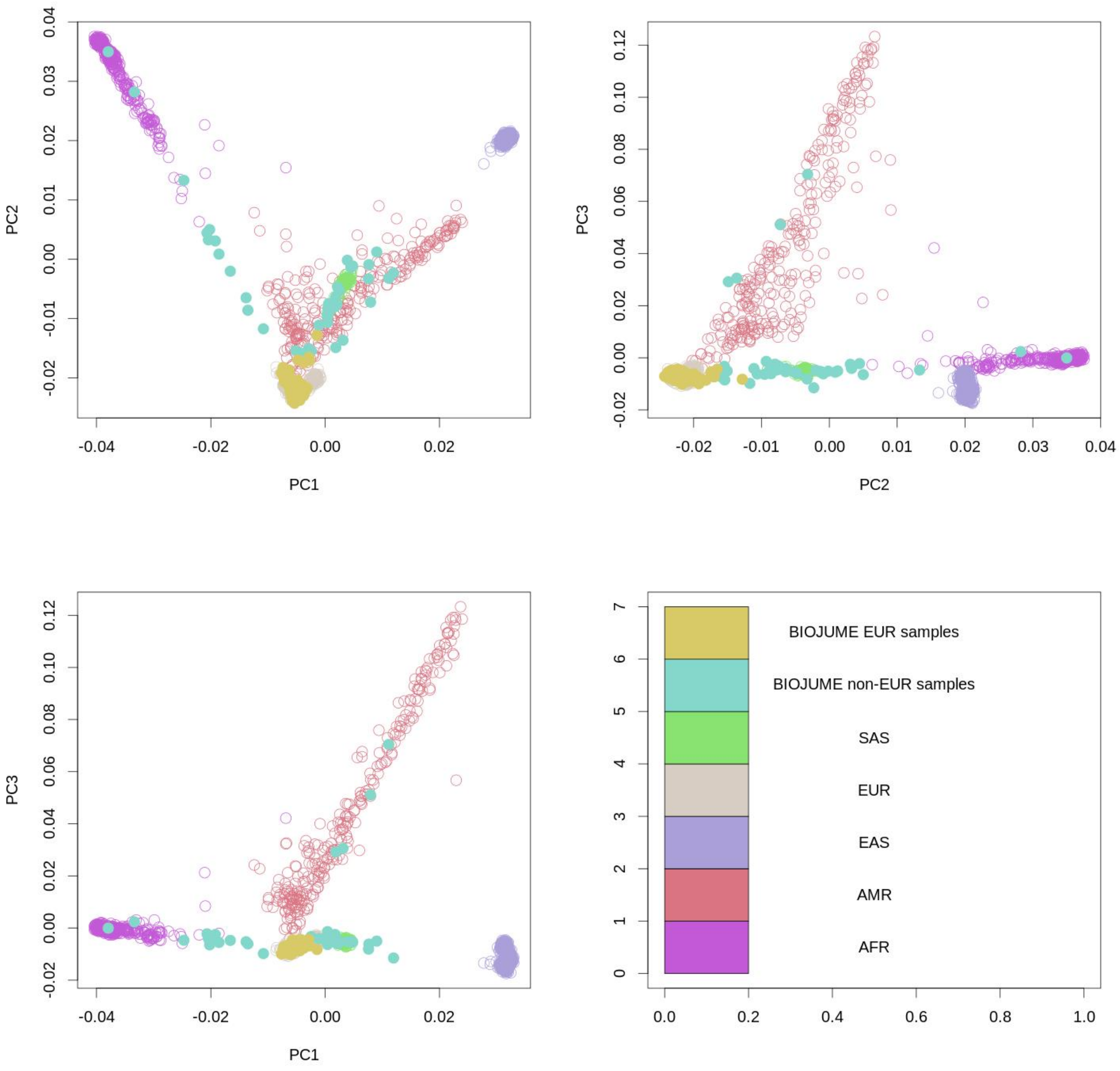

BIOJUME samples who were admixed or AMR were kept if they were within 6 standard deviations from the European cluster. SAS, South Asian; EUR, European; EAS, East Asian; AFR, African; AMR, Ad Mixed American.

Supplementary Figure 7: Colocalization figure from LocusFocus[1] for the *SLCO5A1* gene

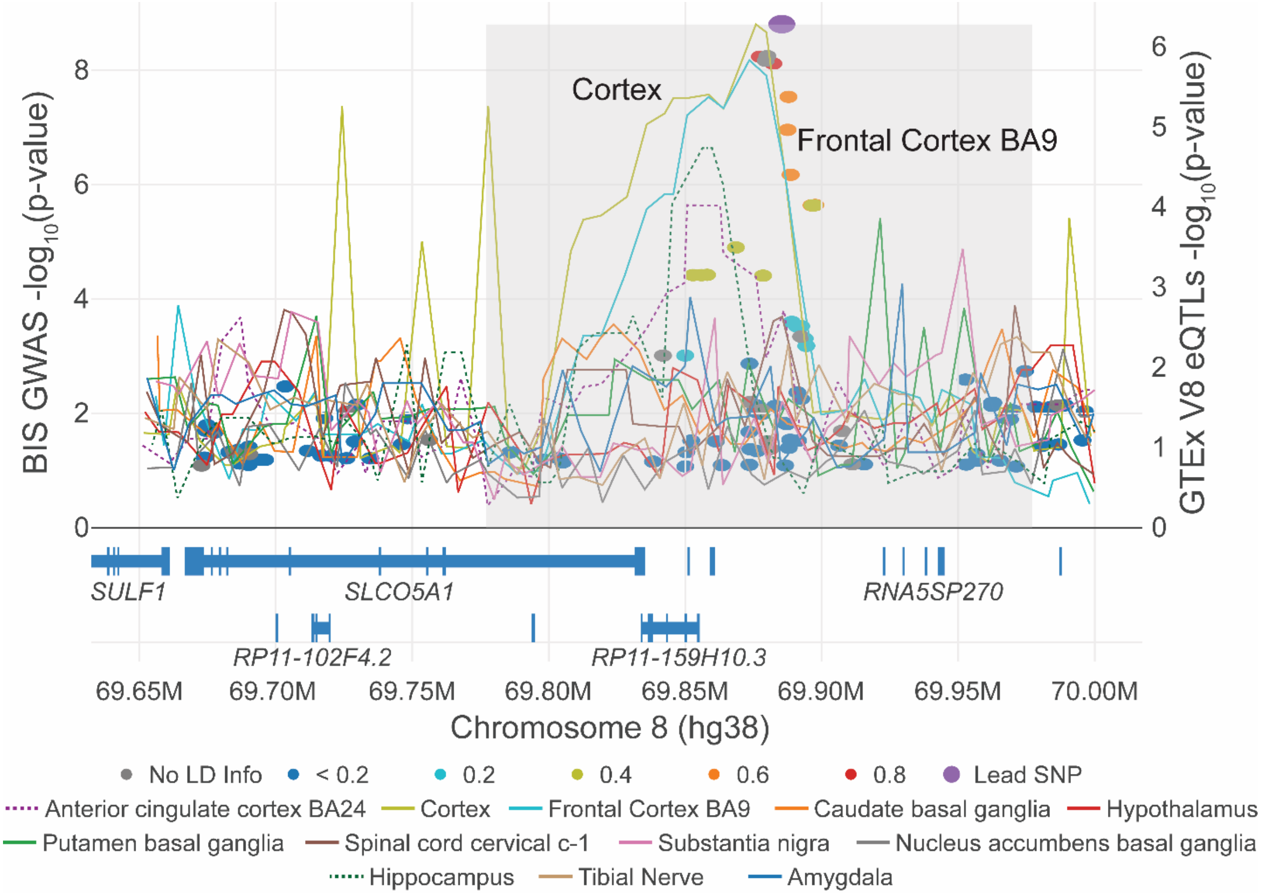

Same as in main Figure 2a, but circles depict the GWAS with BIS-Brief in the European subset (n=324). Colocalization analysis results reveal colocalization with GTEx[2] V8 brain cortex ($-\log_{10}$ Simple Sum P-value (SSP) = 1.36), although this colocalization does not pass the multiple testing significance threshold of $-\log_{10}$SSP=2.0 for testing colocalization across eQTLs from GTEx[2], PsychENCODE[3] and fetal brain eQTLs from O'Brien *et al*.[4]

Supplementary Figure 8. Multiple sequence alignment of putative homologous sequences to human *SLCO5A1* (first row): mouse *Slco5a1* (88.60% identity), *Oatp30B* from fly (*D. melanogaster*; 37.43% identity), and *Oatp26F* from fly (34.63% identity).

```
          1                                                                              80
SLCO5A1   MDEGT-------------------------------------------------------------GLQPGAGEQLEAPA
Slco5a1   MDEGD-------------------------------------------------------------A-QSRAQQQLEAPS
Oatp30B1  MYQMT-------------------------------------------------------------GLYSETN-SGDDS
Oatp30B2  MSAKHIEQYTNPSFEQESDQPPDPLGGVADVGAGNASSSSNGHIANDAGTANRKGHRRQESMYQMTGLYSETN-SGDDS
Oatp26F   MSEAT----------------------------------------------------------------------IELDS

          81                                                                            160
SLCO5A1   TAEAVQERCEPETLRSKSLPVLSSASCRPSLSPTSGDANPAFGCVDSSGHQELKQGPNPLAPSPSAP---------STSA
Slco5a1   SVGAVQEKCESESFRSKSLPVLHSASCRQNFSAGNADGKLALGCNEAPGRQETHLGRNLIVPSPSAP---------STSA
Oatp30B1  SI-DAALDHD-------SQPH--------ATSYLAGDQLPS----------TVGSGPGGGGPSAVADSVTVKCHSRQASA
Oatp30B2  SI-DAALDHD-------SQPH--------ATSYLAGDQLPS----------TVGSGPGGGGPSAVADSVTVKCHSRQASA
Oatp26F   SI-AVAPKCSN---------------------------------------------------------VNNNNSARSSRV

          161                                                                           240
SLCO5A1   GL----GDCNHRVDLSKTFSVSSALAMLQERRCLYVVLTDSRCFLVCMCFLTFIQALMVSGYLSSVITTIERRYSLKSSE
Slco5a1   GTTTELMDCNHRMDPSKTVSVSSTLATLQERRCLYVVLTDSRCFLVCMCFLTFIQALMVSGYLSSVITTIERRYSLKSSE
Oatp30B1  GK----CPADPEEDFDEEQFRSGDCGILNCRPYGIQRFARIKIFVVLLSLLVMMQQALSSGYINSVITTIEKRFEIPSSY
Oatp30B2  GK----CPADPEEDFDEEQFRSGDCGILNCRPYGIQRFARIKIFVVLLSLLVMMQQALSSGYINSVITTIEKRFEIPSSY
Oatp26F   GS----SISGPDS--DAECQRFGWCGW---NPAWLQRFCTAKWALFWLCWGGALQGLIVNGLINVSISTIERRFGLRSRQ

          241                                                                           320
SLCO5A1   SGLLVSCFDIGNLVVVVFVSYFGGRG--RRPLWLAVGGLLIAFGAALFALPHFISPPY--QIQELNASAPNDGLC-----
Slco5a1   SGLLVSCFDIGNLVVVVFVSYFGGRG--RRPLWLAVGGLLIAIGAALFALPHFISPPY--QIQELNASASNDGLC-----
Oatp30B1  SGLIASSYEIGNVITVIFVSYLGSRR--HIPVWIGIGAVIMGIGSLVFMVPHFTGEPN-PGIAIVNKTS--DNICKSALV
Oatp30B2  SGLIASSYEIGNVITVIFVSYLGSRR--HIPVWIGIGAVIMGIGSLVFMVPHFTGEPN-PGIAIVNKTS--DNICKSALV
Oatp26F   MGLVASGYDLASFACLVPVTYYGGRRGASKPRFIAIGLIVMGMGSLVFLLPNFLVGNYRATIAEANVCE-TTGLP-----

          321                                                                           400
SLCO5A1   ----------QGGNSTATLEPPACPKDS---GGNN-HWVYVALFICAQILIGMGSTPIYTLGPTYLDDNVKKENSSLYLA
Slco5a1   ----------QNGNSTAILEPPPCPKDS---GGNS-HWIYVALFVCAQVLIGMGSTPIYTLGPTYLDDNVKKDNASLYLA
Oatp30B1  RDQDMDLGRLSSGLSNQPLAPHTLREDNCLEGKAS-TTGPVLLFVLAQLLLGCGGSPLFTLGTTYVDDHVRTESSSMYIG
Oatp30B2  RDQDMDLGRLSSGLSNQPLAPHTLREDNCLEGKAS-TTGPVLLFVLAQLLLGCGGSPLFTLGTTYVDDHVRTESSSMYIG
Oatp26F   ----------FNSNSSQSMTA--CELNAMGEGQSENLTWTVWLFLAAQLLHGAGASPLFTLGVTYIDENVSKKMSSVYLG
```

```
         401                                                                          480
SLCO5A1  IMYVMGALGPAVGYLLGGLLIGFYVDPR--NP--VHLDQNDPRFIGNWWSGFLLCAIAMFLVIFPMFTFPKKLPPRHKKK
Slco5a1  IMYVMGALGPAVGYLLGGLLIGFYVDPR--NP--VHLDQSDPRFIGNWWSGFLLCAIAMFLVIFPMFTFPKKLPPRHKKK
Oatp30B1 FMYSMGAFGPVVGFLLGAYLLSFHMDSL--SSTTISITPGDRRWVGMWWGGFLLCGVILLVVAVPFFSFPKVLA-REKKK
Oatp30B2 FMYSMGAFGPVVGFLLGAYLLSFHMDSL--SSTTISITPGDRRWVGMWWGGFLLCGVILLVVAVPFFSFPKVLA-REKKK
Oatp26F  IYYTMATVGPAIGYVFGGQLLLIYTDWMTVDPVQLSLTSDSKVWIGAWWLGFIFAAAMCLLIALPIFGYPKLLPGAEKLQ

         481                                                                          560
SLCO5A1  KKKKFSVDAVSD---------DD--------VLKEKS-NNSEQADKKVSSMGFGKDVRDLPRAAVRILSNMTFLFVSLSY
Slco5a1  -KKF-SADVVID---------DD--------IIKEKS-NTSEQMNKKVSPMGFGKNVRDLPRAAVRILSNMTFLFVSLSY
Oatp30B1 IRKSSVVQPVLPNNSRATVATDEMGKVKKLEIVAVTSKEDQSQAPPKVD-TGYGKDIKDIPQSMLRLVKNPVYIVTCLGA
Oatp30B2 IRKSSVVQPVLPNNSRATVATDEMGKVKKLEIVAVTSKEDQSQAPPKVD-TGYGKDIKDIPQSMLRLVKNPVYIVTCLGA
Oatp26F  LERVSEAHATI-------------------------SEAD-----------DSSNVVRGLPRAVLSLLANPTFFFLNLAG

         561                                                                          640
SLCO5A1  TAESAIVTAFITFIPKFIESQFGIPASNASIYTGVIIVPSAGVGIVLGGYIIKKLKLGARESAKLAMICSGVSLLCFSTL
Slco5a1  TAESAIVTAFITFIPKFIESQFGIPASNASIYTGVIIVPSAGVGIVLGGYIIKKLKLGARESAKLAMICSGVSLLCFSTL
Oatp30B1 CMELMIVSGFVVFLPKYLETQFSLGKSQANIFTGSIAVPGACIGIFLGGCILKRFQLKPKGAVQFVLITNVICLACYAML
Oatp30B2 CMELMIVSGFVVFLPKYLETQFSLGKSQANIFTGSIAVPGACIGIFLGGCILKRFQLKPKGAVQFVLITNVICLACYAML
Oatp26F  ATEGLVIAGFAAFLPKQIENQFSISPMYSALVMGLITVPAGGGGTFLGGYLVKKWNLACRGIIKMCLLATTVA-ALFTIC

         641                                                                          720
SLCO5A1  FIVGCESINLGGINIPYTT----GPSLTMPH-RNLTGSCNVNCGCKIHEYEPVCGSDGITYFNPCLAGCVNSGNLSTGIR
Slco5a1  FIVGCESINLGGINIPYTT----GPSLTMPH-RNLTGSCNVNCGCKIHEYEPVCGSDGITYFNPCLAGCINSGNLTTGVR
Oatp30B1 FFLGCDNLKMAGTTIPYYTSNKHGSTLEQPFQVNLTAACNFGCECLTSEVEPVCGNNGLTYFSPCHAGCTAFS--STSNT
Oatp30B2 FFLGCDNLKMAGTTIPYYTSNKHGSTLEQPFQVNLTAACNFGCECLTSEVEPVCGNNGLTYFSPCHAGCTAFS--STSNT
Oatp26F  FLVSCPNPKFAGVTTGYKM----QPSSDSP---ALVASCNSNCGCSRTNYDPICGVDGVMYYSPCYAGCVQEEH-ANSLK

         721                                                                          800
SLCO5A1  NYTECTCVQSRQVITPPTVGQRSQLRVVIVKTYLN---ENGYAVSGKCKRTCNTLIPFLVFLFIVTFITACAQPSAIIVT
Slco5a1  NYTECTCVQSRQVITPPTVGQRSQLRVVIVKTYLN---ENGYAVSGKCKRTCNTLIPFLVFLFIVTFITACAQPSAIIVT
Oatp30B1 NYTNCACVRANISSSIYRGAGGSQAQALSANENFAEVTVVPVATAGPCATPCRTIYPFLILLFFMTFLVASTQMPLLMIV
Oatp30B2 NYTNCACVRANISSSIYRGAGGSQAQALSANENFAEVTVVPVATAGPCATPCRTIYPFLILLFFMTFLVASTQMPLLMIV
Oatp26F  RYHNCSCIEQVGFVD--DGNPSSEAP-----------HFRPDATNRKCDSTCQTLPLFVALCFILMVFTFLATMPALSAT
```

```
          801                                                                            880
SLCO5A1   LRSVEDEERPFALGMQFVLLRTLAYIPTPIYFGAVIDTTCMLWQQECGV-Q-GSCWEYNVTSFRFVYFG-LAAGLKFVGF
Slco5a1   LRSVEDEERPFALGMQFVLLRTLAYIPTPIYFGAVIDTTCMLWQQECGV-Q-GSCWEYNVTSFRFVYFG-LAAGLKFVGF
Oatp30B1  LRSVSEEERSFALGMQFVIFRLFGYIPAPILFGNLIDSTCILWKSSCGE-KGGRCLIYDIEKFRYKYVG-LCASVKLIAL
Oatp30B2  LRSVSEEERSFALGMQFVIFRLFGYIPAPILFGNLIDSTCILWKSSCGE-KGGRCLIYDIEKFRYKYVG-LCASVKLIAL
Oatp26F   LRCVQDDQRSFALGLQWIKVRLLGTIPAPLIFGALIDESCILWQESCDKDAGGACLVYDNFYI-SRYMWLLALICKLGSV

          881                                                                            960
SLCO5A1   IFIFLAWYSIKYKEDGLQRRRQREFPLST---------------------------------------------------
Slco5a1   IFIFLAWYSIKYKEDGLQRRRCREFPLST---------------------------------------------------
Oatp30B1  VIFMVDWWLVRRRKQ-LEKM----KPLNASDPIIGSIISLDKLFEEKLSGAEPSTAFVGGGGELIIPTDILRHSRNDSRT
Oatp30B2  VIFMVDWWLVRRRKQ-LEKM----KPLNASDPIIGSIISLDKLFEEKLSGAEPSTAFVGGGGELIIPTDILRHSRNDSRT
Oatp26F   VFFACAWWFYVPP------S----KPLNA---------------------------------------------------

          961                                                                           1040
SLCO5A1   ---------------VSERVGHP-D----NARTRSCPAF-----------------------------------------
Slco5a1   ---------------VSEQVGQP-SKAEKYSRTTSCPAF-----------------------------------------
Oatp30B1  MHMDYCYDKCGRVVTPANTCNQPQTKSKKHFRSASCDVKMIKSFARDHSSSSGPADAAGQDAVGASTKYKNLKKFQAHTR
Oatp30B2  MHMDYCYDKCGRVVTPANTCNQPQTKSKKHFRSASCDVKMIKSFARDHSSSSGPADAAGQDAVGASTKYKNLKKFQAHTR
Oatp26F   --------------------------------------------------------------------------------

          1041                                                                          1120
SLCO5A1   ----------------------------STQGEF----------------------------------------------
Slco5a1   ----------------------------STQGEV----------------------------------------------
Oatp30B1  NHSTDLHDPSQPIRYIQNQLRPQDCPEEDDDEELTTGCGHFVKKHSRNHSYDQIYMPNNIRFDADFLRHPHSHHNPKKNV
Oatp30B2  NHSTDLHDPSQPIRYIQNQLRPQDCPEEDDDEELTTGCGHFVKKHSRNHSYDQIYMPNNIRFDADFLRHPHSHHNPKKNV
Oatp26F   --------------------------------------------------------------------------------

          1121                                                                          1200
SLCO5A1   -------------HE----------------------ETGLQKGIQCAAQTYP----------------------GPFPE
Slco5a1   -------------HE----------------------ETALQKGFPCTTQTYP----------------------GPFSE
Oatp30B1  NVLKNVVSDVGKLKNSNEIEAGGAGSRGHSRNNSKDLNTKISSATPASGQVVTDASTTGLSVLRHRRTNSKDLNYQVLPE
Oatp30B2  NVLKNVVSDVGKLKNSNEIEAGGAGSRGHSRNNSKDLNTKISSATPASGQVVTDASTTGLSVLRHRRTNSKDLNYQVLPE
Oatp26F   --------------------------------------------------------------------------------
```

```
           1201                                               1251
SLCO5A1    AISSSADPG------------------------------LEESPAALEPPS
Slco5a1    AVSSSADPG------------------------------QDESP---DAAL
Oatp30B1   SAASSSVSGHAHPQHTRNTSHHKIQIDDDRNELINDNDDEEEER---SACA
Oatp30B2   SAASSSVSGHAHPQHTRNTSHHKIQIDDDRNELINDNDDEEEER---SACA
Oatp26F    ----------------------------------------NGK---EDVN
```

Alignment confidence (per residue): high **9876543210** low

Domain	Corresponding residues in human *SLCO5A1* (source: Pfam)
**MFS_1**	135-529
**OATP**	129-734
**Kazal**	549-603

Overlapping domain regions are underlined.

The multiple sequence alignment was performed using MUSCLE5.[5] All sequences have a Kazal-like domain (shown in red).

Supplementary Figure 9. (a) ***Oatp30B*** **mRNA levels as measured by qPCR (b) Reduced lifespan in flies with** ***Oatp30B*** **knock-down. (c) Unchanged speed during action. (d) Unaffected duration of single action bouts. (e) Unaffected rest interval in between single action bouts.**

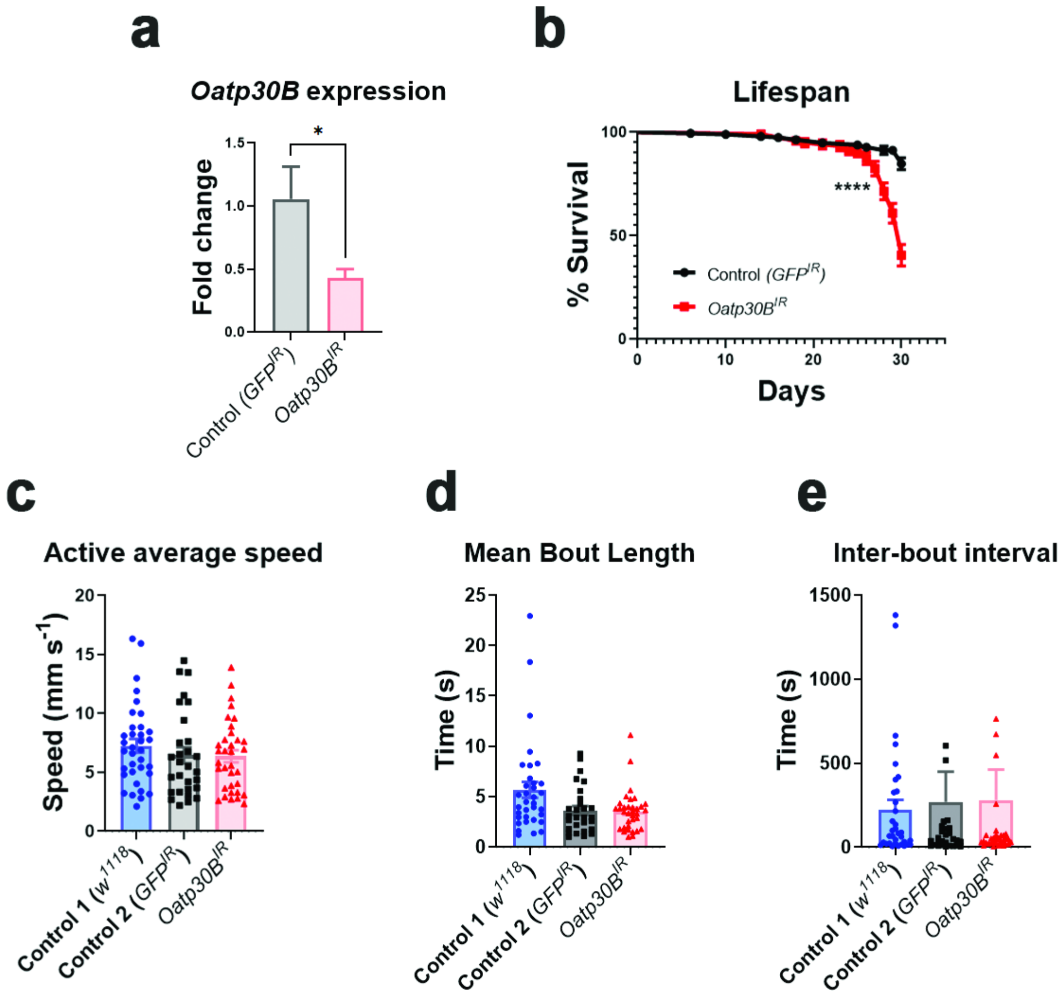

a: The *UAS-Oatp30B*^{IR} (GD12775) transgenic or the control *UAS-GFP*^{IR} were driven with *Tub-Gal4* and *Ubi-Gal80ts*. The graph reports data from 3 biological samples (n=10 flies, both males and females) and 5 technical replicates. Mean +/- SEM * P<0.05, Unpaired t-test, one-tailed.

b: The *UAS-Oatp30B*^{IR} (GD12775) transgenic or the control *UAS-GFP*^{IR} were driven with *nSyb-Gal4* and *Ubi-Gal80ts*. Percent +/- SE **** P<0.0001, Log-rank (Mantel-Cox) test, $\square^2$ 51.74 for 1 df, n=119-194.

c: The *UAS-Oatp30B*^{IR} (GD12775) transgenic or the control *UAS-GFP*^{IR} were driven with *nSyb-Gal4* and *Ubi-Gal80ts*. The *w*^{1118} strain is a control for the genetic background in absence of transgenes.

d: Flies as in c.

e: Flies as in c.

Supplementary Figure 10. BIS-Brief score vs. ADHD PRS deciles

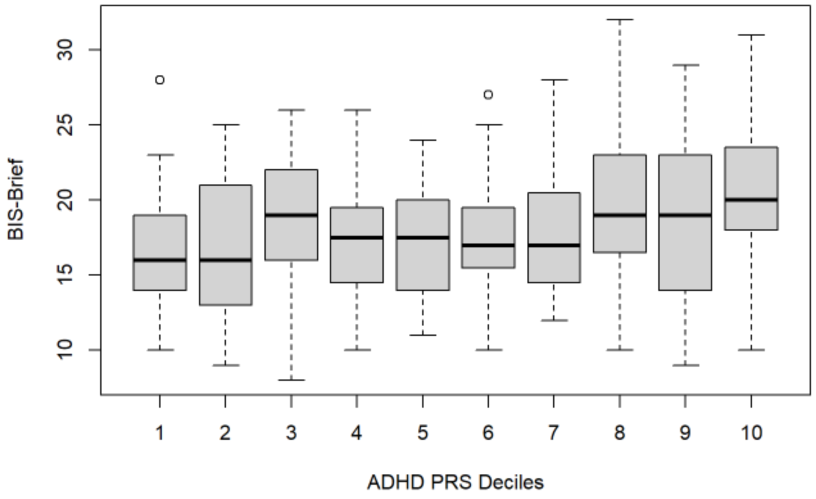

The center lines represent the 50th percentile (median) and the bounds of the boxes are the 75th and 25th percentiles (interquartile range) with the whiskers being the largest value within 1.5 times the interquartile range above the 75th percentile and smallest values within 1.5 times the interquartile range below the 25th percentile.

Supplementary Tables

Supplementary Table 1: Methods applied for the computation of PRS.

	Summary statistics				BIOJUME	Clumping + Thresholding	
**Disease**	**Study**	**Sample size**	**Ethnicity**	**Variant filtering**	**Variant Filtering**	**Clumping**	**p-value Threshold**
**ADHD**	Demontis et al., 2019[6]	19,099 cases & 34,194 controls	European	MAF ≤0·05<br>r^2 ≤0·8<br>Indels<br>Ambiguous SNPs<br>Multi-allelic SNPs<br>MHC region	r^2 ≤0·5	r^2: 0·3<br>radius: 500 kb	0·1
**Risk Taking**	UK Biobank[7]	348,549	European	MAF ≤0·05<br>Info ≤0·8<br>Indels<br>Ambiguous SNPs<br>Multi-allelic SNPs	r2 ≤0·5	r2: 0·1<br>radius: 500 kb	0·05*
**Bipolar Disorder**	Stahl et al., 2019[8]	20,352 cases and 31,358 controls	European	MAF ≤0·05<br>Info ≤0·9<br>Indels<br>Ambiguous SNPs<br>Multi-allelic SNPs<br>MHC region except for the most significant associated SNP (rs36034627, Chr6:27,269,584, T>G)	r^2 ≤0·5	r^2: 0·1<br>radius: 500 kb	0·2
**Generalized epilepsy**	ILAE Consortium, 2018[9]	15,212 cases & 29,677 controls	Majority European	MAF ≤0·02<br>r^2 ≤0·3<br>Ambiguous SNPs	r^2 ≤0·5	r^2: 0·1<br>radius: 500 kb	0·5
**Focal epilepsy**	ILAE Consortium, 2018[9]	15,212 cases & 29,677 controls	Majority European	MAF ≤0·02<br>r^2 ≤0·3<br>Ambiguous SNPs	r^2 ≤0·5	r^2: 0·1<br>radius: 500 kb	0·5

Clumping and thresholding were used to calculate ADHD, risk taking, bipolar disorder, generalized epilepsy, and focal epilepsy PRS in individuals of European ancestry using PLINK v1·9.[10] PRS values were generated by weighting selected SNPs after clumping and thresholding by the additive scale effect (log_{10}(OR)/Beta), and then summing over the variants.

For generalized and focal epilepsy PRSs, we tested 10 different p-value thresholds (5E-8, 1E-6, 1E-4, 1E-3, 1E-2, 0·05, 0·1, 0·2, 0·5, 1), and 0.05 explained the highest proportion of variation in BIS-Brief score (R^2 = 0·016).

MHC region: The major histocompatibility complex region, chr6:25-34Mbp
ILAE: International League Against Epilepsy
The methods applied to calculated generalized and focal epilepsy PRS are based on Leu et al. 2019.[11]

Supplementary Table 2: Univariate analysis with BIS-Brief

Variable	Beta	Standard Error	P-value
Sex	-1·36	0·51	$8{\cdot}0 \times 10^{-3}$
Age at consent	0·042	0·028	0·13
Myoclonus or absence seizure frequency	1·09	0·25	$2{\cdot}2 \times 10^{-5}$
PC1	3·96	5·05	0·43
PC2	-4·48	5·42	0·41
PC3	4·30	4·39	0·33
Cohort 2	1·41	0·78	0·071
Cohort 3	1·49	0·92	0·10
Cohort 4	1·66	0·79	0·035

Linear regression was used to test association of each variable with BIS-Brief in 324 European individuals with JME. We have previously shown the association of sex and seizure frequency with BIS-Brief.[12] Seizure frequency was used as a marker of controlled seizures and was defined as missing if there was no reported myoclonus frequency; otherwise, it was the maximum observed frequency for myoclonus or absence seizure as follows: daily seizures=3; weekly=2; less than weekly=1; none (currently or ever)=0. Cohort refers to the genotyping batch; samples recruited were genotyped in four batches at different time points.

Supplementary Table 3: Association of top BIS-Brief GWAS SNPs with inverse rank normal transformed BIS-Brief (N = 324)

Variant ID	Position (hg38)	Alleles	Beta	SE	P-value
rs73293634	chr8:69,884,968	G/T	1.2	0.2	3.1E-8
rs75042057	chr10:34,202,650	T/G	1.6	0.3	1.4E-7

Linear regression was used to test association of each SNP with inverse rank normal transformed BIS-Brief. Sex, genotyping batch, age at consent, first 3 PCs, and the frequency of myoclonus or absence seizures were included as covariates in the model.

Supplementary Table 4: Association of ADHD, bipolar disorder, and focal and general epilepsy PRS with BIS-Brief

PRS	β	SE	P
**ADHD**	0.09	0.03	1.60E-3
**Risk Taking**	2.14	0.90	1.83E-2
**Bipolar Disorder**	0.07	0.04	0.080
**Generalized Epilepsy PRS**	-0.03	0.03	0.33
**Focal Epilepsy PRS**	0.002	0.045	0.96

Association of each PRS with BIS-Brief was tested using linear regression with age, sex, and frequency of absence/myoclonic seizure as covariates in the model.

References

1 Panjwani, N. *et al.* LocusFocus: Web-based colocalization for the annotation and functional follow-up of GWAS. *PLoS Comput Biol* **16**, e1008336, doi:10.1371/journal.pcbi.1008336 (2020).

2 GTEx Consortium. The Genotype-Tissue Expression (GTEx) project. *Nat Genet* **45**, 580-585, doi:10.1038/ng.2653 (2013).

3 Wang, D. *et al.* Comprehensive functional genomic resource and integrative model for the human brain. *Science* **362**, doi:10.1126/science.aat8464 (2018).

4 O'Brien, H. E. *et al.* Expression quantitative trait loci in the developing human brain and their enrichment in neuropsychiatric disorders. *Genome Biol* **19**, 194, doi:10.1186/s13059-018-1567-1 (2018).

5 Edgar, R. C. MUSCLE: multiple sequence alignment with high accuracy and high throughput. *Nucleic Acids Res* **32**, 1792-1797, doi:10.1093/nar/gkh340 (2004).

6 Demontis, D. *et al.* Discovery of the first genome-wide significant risk loci for attention deficit/hyperactivity disorder. *Nat Genet* **51**, 63-75, doi:10.1038/s41588-018-0269-7 (2019).

7 *Neale's Lab UK Biobank GWAS Results Round 2 (Imputed v3 - File Manifest Release 20180731)*, <http://www.nealelab.is/uk-biobank> (

8 Stahl, E. A. *et al.* Genome-wide association study identifies 30 loci associated with bipolar disorder. *Nat Genet* **51**, 793-803, doi:10.1038/s41588-019-0397-8 (2019).

9 International League Against Epilepsy Consortium on Complex, E. Genome-wide mega-analysis identifies 16 loci and highlights diverse biological mechanisms in the common epilepsies. *Nat Commun* **9**, 5269, doi:10.1038/s41467-018-07524-z (2018).

10 Purcell, S. *et al.* PLINK: a tool set for whole-genome association and population-based linkage analyses. *Am J Hum Genet* **81**, 559-575, doi:10.1086/519795 (2007).

11 Leu, C. *et al.* Polygenic burden in focal and generalized epilepsies. *Brain* **142**, 3473-3481, doi:10.1093/brain/awz292 (2019).

12 Shakeshaft, A. *et al.* Sex-specific disease modifiers in juvenile myoclonic epilepsy. *Sci Rep* **12**, 2785, doi:10.1038/s41598-022-06324-2 (2022).
