# Supplementary material for: SLCO5A1 and synaptic assembly genes contribute to impulsivity in juvenile myoclonic epilepsy
Source: NPJ Genom Med. 2023 Sep 28;8:28. doi: 10.1038/s41525-023-00370-z (PMC10539321; doi:10.1038/s41525-023-00370-z)
Supplement: Supplementary file 2 — Reporting Summary [file 41525_2023_370_MOESM2_ESM.pdf]

nature portfolio

Corresponding author(s): Lisa J. Strug & Deb K. Pal

Last updated by author(s): Jun 22, 2023

Reporting Summary

Nature Portfolio wishes to improve the reproducibility of the work that we publish. This form provides structure for consistency and transparency in reporting. For further information on Nature Portfolio policies, see our Editorial Policies and the Editorial Policy Checklist.

Statistics

For all statistical analyses, confirm that the following items are present in the figure legend, table legend, main text, or Methods section.

n/a	Confirmed	
☐	☒	The exact sample size (*n*) for each experimental group/condition, given as a discrete number and unit of measurement
☐	☒	A statement on whether measurements were taken from distinct samples or whether the same sample was measured repeatedly
☐	☒	The statistical test(s) used AND whether they are one- or two-sided<br>*Only common tests should be described solely by name; describe more complex techniques in the Methods section.*
☐	☒	A description of all covariates tested
☐	☒	A description of any assumptions or corrections, such as tests of normality and adjustment for multiple comparisons
☐	☒	A full description of the statistical parameters including central tendency (e.g. means) or other basic estimates (e.g. regression coefficient) AND variation (e.g. standard deviation) or associated estimates of uncertainty (e.g. confidence intervals)
☐	☒	For null hypothesis testing, the test statistic (e.g. *F*, *t*, *r*) with confidence intervals, effect sizes, degrees of freedom and *P* value noted<br>*Give P values as exact values whenever suitable.*
☒	☐	For Bayesian analysis, information on the choice of priors and Markov chain Monte Carlo settings
☒	☐	For hierarchical and complex designs, identification of the appropriate level for tests and full reporting of outcomes
☐	☒	Estimates of effect sizes (e.g. Cohen's *d*, Pearson's *r*), indicating how they were calculated

Our web collection on statistics for biologists contains articles on many of the points above.

Software and code

Policy information about availability of computer code

Data collection	No software was used.
Data analysis	PLINK v1·90b6·18<br>KING v.2·2·4<br>PEDSTATS 0·6·12<br>PC-AiR in the GENESIS v2·16·0<br>McCarthy Tools v4·3·0<br>TOPMED imputation server<br>Eagle v2·4<br>minimac v4 v1·0·2<br>Ensembl Variant Effect Predictor (v94)<br>LocusFocus v1.4.9 alpha<br>DART software<br>The details of where each software was used are provided in the manuscript.

For manuscripts utilizing custom algorithms or software that are central to the research but not yet described in published literature, software must be made available to editors and reviewers. We strongly encourage code deposition in a community repository (e.g. GitHub). See the Nature Portfolio guidelines for submitting code & software for further information.

Data

Policy information about availability of data

All manuscripts must include a data availability statement. This statement should provide the following information, where applicable:

- Accession codes, unique identifiers, or web links for publicly available datasets
- A description of any restrictions on data availability
- For clinical datasets or third party data, please ensure that the statement adheres to our policy

eQTL data are available for download from GTEx (https://gtexportal.org/home), PsychENCODE (http://resource.psychencode.org/), and fetal brains (https://doi.org/10.6084/m9.figshare.6881825). GWAS summary statistics for this study are available for download from our website (https://lab.research.sickkids.ca/strug/softwareandresources/).

Research involving human participants, their data, or biological material

Policy information about studies with human participants or human data. See also policy information about sex, gender (identity/presentation), and sexual orientation and race, ethnicity and racism.

Reporting on sex and gender	Gender data was not collected in this study. Sex was determined by genetic data.
Reporting on race, ethnicity, or other socially relevant groupings	Ethnicity was determined by genetic PCA analysis. The analysis was performed in European subjects and in all ethnicities combined. Population stratification was included as covariate in the combined analysis.
Population characteristics	Sex, genotyping batch, age at consent, population stratification, and the frequency of myoclonus or absence seizures were included as covariates in the model.
Recruitment	We collected cross-sectional clinical and genetic data from the Biology of Juvenile Myoclonic Epilepsy (BIOJUME) consortium study, which focuses on gathering cases with JME. BIOJUME is a study across 50 sites in 10 countries. All participants' medical history was reviewed by a phenotyping committee to validate the diagnosis of JME.
Ethics oversight	Ethical approval was obtained from UK Health Research Authority: South Central Oxford C Research Ethics Committee (16/SC/0266) and all other collaborating sites. The SickKids Research Ethics Committee of The Hospital for Sick Children (1000033784) also gave ethical approval for this work.

Note that full information on the approval of the study protocol must also be provided in the manuscript.

Field-specific reporting

Please select the one below that is the best fit for your research. If you are not sure, read the appropriate sections before making your selection.

☒ Life sciences ☐ Behavioural & social sciences ☐ Ecological, evolutionary & environmental sciences

For a reference copy of the document with all sections, see nature.com/documents/nr-reporting-summary-flat.pdf

Life sciences study design

All studies must disclose on these points even when the disclosure is negative.

Sample size	The sample size was sufficient to detect genetic variants that explain 12% of the variance in the BIS score with 80% power after adjusting for multiple hypothesis testing at the genome-wide significance level.
Data exclusions	Inclusion criteria were based on Avignon Class II consensus criteria for JME diagnosis:<br>• Age of myoclonus onset 6-25 years<br>• Myoclonic seizures with predominant/exclusive earlymorning pattern involving upper extremities<br>• Electroencephalogram (EEG) showing interictal generalized spikes and/or polyspike and waves on a normal background<br>• Current age between 6-55 years.<br>Exclusion criteria:<br>• Myoclonic seizures only associated with carbamazepine or lamotrigine therapy<br>• EEG showing predominant focal interictal epileptiform discharges or abnormal background<br>• Evidence of progressive or symptomatic myoclonus epilepsy or focal seizures<br>• Global learning disability<br>• Dysmorphic syndrome<br>• Unable to provide informed consent.
Replication	Drosophila models were performed in 3 experiments including up to 12 flies per genotype. The results were consistent in the 3 experiments.

Randomization	NA
Blinding	NA

Reporting for specific materials, systems and methods

We require information from authors about some types of materials, experimental systems and methods used in many studies. Here, indicate whether each material, system or method listed is relevant to your study. If you are not sure if a list item applies to your research, read the appropriate section before selecting a response.

Materials & experimental systems

n/a	Involved in the study	
☒	☐	Antibodies
☒	☐	Eukaryotic cell lines
☒	☐	Palaeontology and archaeology
☐	☒	Animals and other organisms
☐	☒	Clinical data
☒	☐	Dual use research of concern
☒	☐	Plants

Methods

n/a	Involved in the study	
☒	☐	ChIP-seq
☒	☐	Flow cytometry
☒	☐	MRI-based neuroimaging

Animals and other research organisms

Policy information about studies involving animals; ARRIVE guidelines recommended for reporting animal research, and Sex and Gender in Research

Laboratory animals	Drosophila
Wild animals	The study did not involve wild animals.
Reporting on sex	Female and male flies were pooled together and equally distributed within vials.
Field-collected samples	The study did not involved samples collected from field.
Ethics oversight	NA

Note that full information on the approval of the study protocol must also be provided in the manuscript.

Clinical data

Policy information about clinical studies

All manuscripts should comply with the ICMJE guidelines for publication of clinical research and a completed CONSORT checklist must be included with all submissions.

Clinical trial registration	The study is not clinical trial.
Study protocol	The study is not clinical trial.
Data collection	We collected cross-sectional data as part of the Biology of Juvenile Myoclonic Epilepsy (BIOJUME) genome-wide association consortium study across 50 sites in 10 countries. Research staff collected clinical data face-to-face in the form of a structured questionnaire, augmented by clinical records, EEG reports, and digital EEGs. The dataset included general demographics and health information, epilepsy history including seizure types, seizure frequency, drug/lifestyle interventions, and BIS-brief.
Outcomes	NA
